# The Effects of Mind–Body Exercises on Chronic Spinal Pain Outcomes: A Synthesis Based on 72 Meta-Analyses

**DOI:** 10.3390/ijerph191912062

**Published:** 2022-09-23

**Authors:** Javier Martinez-Calderon, Maria de-la-Casa-Almeida, Javier Matias-Soto

**Affiliations:** 1 Department of Physiotherapy, Faculty of Nursing, Physiotherapy and Podiatry, University of Sevilla, Avicena s/n, 41009 Sevilla, Spain; 2 Uncertainty, Mindfulness, Self, Spirituality (UMSS) Research Group, University of Seville, 41004 Seville, Spain; 3 Department of Physical Therapy, Faculty of Health Sciences, University of Malaga, Arquitecto Francisco Peñalosa, 3, 29071 Malaga, Spain

**Keywords:** chronic low back pain, chronic neck pain, chronic spinal pain, qigong, tai chi, yoga

## Abstract

An umbrella review of systematic reviews with a meta-analysis was developed to summarize the evidence on the effectiveness of qigong, tai chi, and yoga in chronic spinal pain outcomes. The CINAHL, Cochrane Library, Embase, PsycINFO, PubMed and SPORTDiscus databases were searched. Pain, psychological factors, and quality of life (QOL) were the outcomes of interest. The methodological quality of the reviews was evaluated using the AMSTAR-2 tool. The overlap was calculated using the corrected covered area. A total of 72 meta-analyses drawn from 20 systematic reviews were included and often were rated at a critically low quality. The effects of qigong on chronic low back and neck pain (CLBP and CNP, respectively) were inconsistent, although it improved the physical component of QOL after 12 weeks for CNP. Tai chi was superior to the controls in reducing CLBP; no reviews of interest were found on CNP. Yoga was superior to multiple controls in reducing CLBP, but no relevant effects on depression or QOL were found. QOL, anxiety, depression, and general mood improved with yoga for CNP. Inconsistencies arose related to yoga and CNP. Our findings mainly supported the potential effects of yoga and tai chi on pain-related outcomes, psychological factors, and QOL in populations with CLBP and NP. Clinical and methodological considerations were discussed.

## 1. Introduction

Chronic spinal pain is very prevalent and burdensome [1,2,3,4], as low back pain is the leading global cause of years of disability [5]. The Global Spine Care Initiative proposed that people with spinal problems need to empower and improve their self-states (e.g., self-efficacy) to develop autonomous and proactive strategies related to their care [6]. However, chronic spinal pain implies a complex interaction between multiple biopsychosocial factors [7,8], and many people often experience an internal battle to maintain their previous selves [9] and difficulties in integrating healthy activities into their daily lives [10].

Exercise is key for people with chronic musculoskeletal pain [11], and nontraditional exercises such as qigong, tai chi, and yoga produce not only physical and physiological benefits [12,13,14,15], but also psychological and spiritual well-being [16,17,18,19]. These mindful exercises help people connect with themselves using meditative and deep-breathing strategies, promoting greater self-regulation skills [16,20,21]. Previous overviews of systematic reviews evaluated the role of these mindful approaches in health [22,23,24,25,26,27]. However, a large number of systematic reviews on yoga, tai chi, or qigong in chronic spinal pain have not been previously evaluated [28,29,30,31,32,33,34,35,36,37]; the methodological quality was scarcely analyzed; and the potential overlap between the trials explored has not yet been tested.

Therefore, this umbrella review aimed to summarize the following research question: are qigong, tai chi, and yoga more effective than any type of control group in modulating pain, psychological factors, and quality of life in people with chronic spinal pain based on systematic reviews with a meta-analysis?

## 2. Materials and Methods

This umbrella review followed the Preferred Reporting Items for Overviews of Systematic Reviews (PRIO-harms) [38]. The review protocol was registered on the Open Science Framework: https://doi.org/10.17605/OSF.IO/A6GBT.

### 2.1. Deviations from the Protocol

Some information that was published in our review protocol was not included in this umbrella review. The mean age and sex distribution was not extracted from the included reviews. The Grading of Recommendations, Assessment, Development, and Evaluations (GRADE) approach [39] was not applied due to the presence of overlap between reviews.

### 2.2. Data Sources and Search Strategy

A researcher (MCA) searched in six electronic databases from database inception until 6 March 2022: CINAHL (via EBSCOhost), the Cochrane Library, Embase, PsycINFO (via ProQuest), PubMed, and SPORTDiscus (via EBSCOhost). A PubMed search strategy was built and implemented in other databases when possible. Ethnicity, gender, or setting restrictions were not imposed. Supplementary File S1 shows all search strategies.

### 2.3. Eligibility Criteria

The patient, intervention, comparison, and outcome (PICO) framework [40] was used to include systematic reviews with a meta-analysis that were written in English or Spanish and included adults with chronic spinal pain. The interventions of interest were any style of qigong, tai chi, or yoga. There were no restrictions on the control group. Pain, psychological factors, and quality of life were our outcomes of interest. Only systematic reviews that meta-analyzed randomized trials were considered. We decided that a meta-analysis would only include whether two trials were at least meta-analyzed.

We excluded reviews that were: (I) abbreviated reports of those Cochrane reviews that were included in our umbrella review; (II) network meta-analyses; (III) reviews whose topic was pregnancy-related low back pain; (IV) previous versions of those Cochrane reviews that were included in our umbrella review; (V) conditions or interventions of interest that were not meta-analyzed separately from other conditions or interventions; (VI) reviews that mixed primary and secondary research (e.g., systematic reviews and trials); (VII) overviews of reviews; (VIII) review protocols; and (IX) theses and conference abstracts.

### 2.4. Study Selection

Duplicates were removed using Mendeley Desktop Citation Management Software v1.19.8 and manually checked [41]. A researcher (J.M.C.) screened the titles and abstracts of each reference. Only references that presented the words “systematic review” and/or “meta-analysis” in the title were evaluated.

The same researcher evaluated a total of 174 full texts. The list of references for each review that met our inclusion criteria was manually checked. The reviews included in those overviews excluded in our last screening process were also checked. When necessary, a consensus was reached between all authors. Emails were sent to request additional information when data were unavailable. A reminder was sent two weeks after the first email.

### 2.5. Methodological Quality

The AMSTAR 2 tool [42] was used by two independent reviewers (J.M.S. and M.C.A.) to determine the methodological quality of the included reviews. The instrument consisted of 16 items that could be rated as yes, partially yes, or no [42]. Seven items (2, 4, 7, 9, 11, 13, 15) were proposed as critical, affecting the overall confidence of each review [42]. Overall confidence could be rated as high (no weaknesses or one noncritical item), moderate (more than one noncritical item), low (one critical item with or without noncritical items), or critically low (more than one critical item with or without noncritical items) [42]. The consensus solved any disagreements between both researchers.

### 2.6. Data Extraction and Synthesis

A researcher (J.M.S.) extracted the following information from each included review: the first author and year of publication, the quality assessment and/or risk of bias tool used, the number of randomized trials that were meta-analyzed and satisfied our criteria, the number of participants in these trials, the experimental and control interventions, the effect sizes with their interval confidence, *p*-values, and heterogeneity values (I-square). Our first step was to extract the effect sizes from those meta-analyses that evaluated an overall effect. When some of them did not satisfy our criteria, we decided to extract the effect sizes from subgroup analyses prioritized in the following order: (I) time point effects; (II) clinical condition; (III) experimental group; and (IV) control group. Regarding quality of life, we decided to extract the effect sizes of their most common domains (physical functioning and mental health) when the measure “overall quality of life” was unavailable or did not meet our criteria. Finally, when the goal of a determined review was not chronic low back or neck pain but instead low back or neck pain in general (without limiting pain duration), we decided to screen the table of the characteristics of the included trials in that review or, in the case of unreported information, we checked the original trials. Meta-analyses were excluded when we could not ensure the presence of chronicity for a specific trial (e.g., unreported data or language limitation (such as trials published in Chinese languages)) or when the sample included chronic and nonchronic spinal pain.

The results were narratively divided according to the type of clinical condition; that is, chronic low back pain or chronic neck pain. Subsequently, each section was separated by the type of experimental group (qigong, tai chi, or yoga). Additionally, tables were developed to show the main characteristics and the effect sizes of the included reviews.

### 2.7. Overlapping between Reviews

Citation matrices were developed and the corrected covered area (CCA) [43] was calculated to detect if there was overlapping between the included reviews. The CCA represented the area that was covered after removing each trial the first time it was counted. The overlap could be slight (CCA < 5%), moderate (CCA from 6% to 10%), high (CCA from 11% to 15%), or very high (CCA < 15%) [43].

### 2.8. Co-Occurrence Analysis

The software VOSviewer 1.6.18 (www.vosviewer.com) was applied to develop maps using bibliographic data. This software can detect patterns of terms in a topic. The co-occurrence analysis was based on the keywords reported by each included review through a full counting method. This approach is useful to readers in understanding possible interrelations between reviews that cover the same or similar topics.

## 3. Results

**Figure 1:**
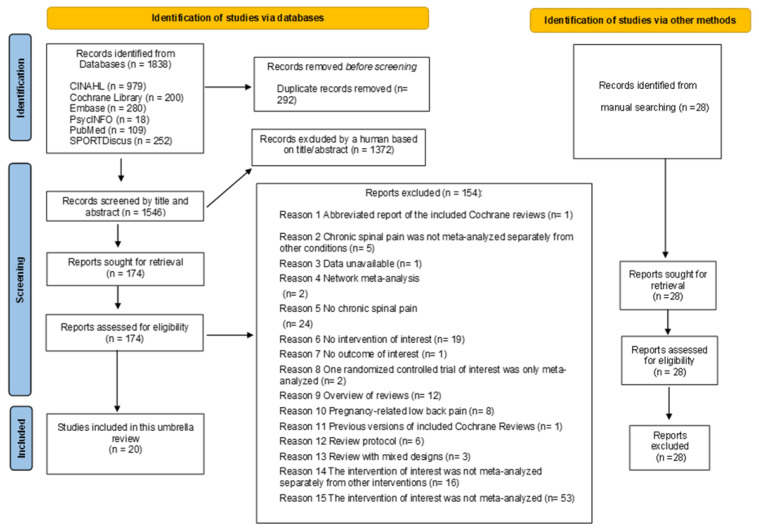
Flow diagram.

The electronic databases retrieved 1838 references. A total of 1546 titles and abstracts were checked after removing duplicates. Finally, 174 full texts were evaluated, of which 154 were excluded (Supplementary File S2). Twenty systematic reviews met our inclusion criteria [13,28,29,30,31,32,33,34,35,36,37,44,45,46,47,48,49,50,51,52] (Figure 1). However, some meta-analyses performed in these reviews were not included in our umbrella. Supplementary File S3 lists the reasons for these exclusions. Twenty-eight additional reviews were found during manual searches (Figure 1). However, none of them met our inclusion criteria. References to these reviews are shown in Supplementary File S4. The included reviews recovered 44 original trials without double counting (Supplementary File S5). These reviews analyzed chronic low back and neck pain populations. Chronic low back pain was the most common spinal disorder. Yoga was commonly evaluated among the included reviews. Methodological quality was often assessed using the PEDro scale [53] or the Cochrane Risk of Bias tool [54]. The overall certainty of the evidence using the GRADE approach [39] was only applied to 30% of the included reviews.

### 3.1. Co-Occurrence Analysis

**Figure 2:**
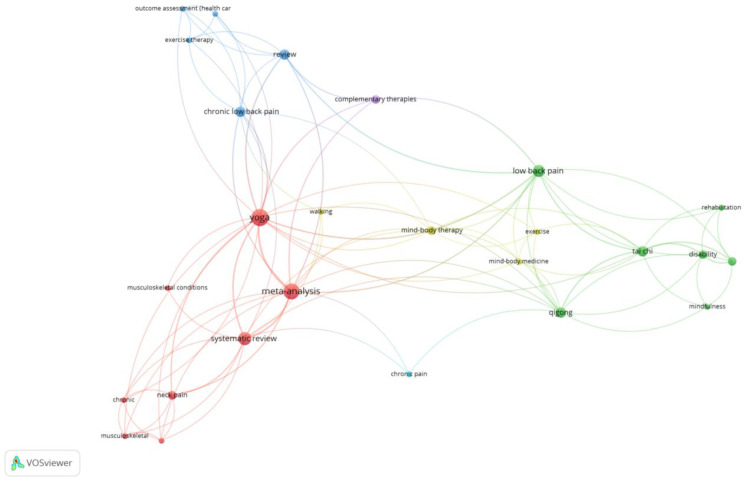
Network Visualization.

**Figure 3:**
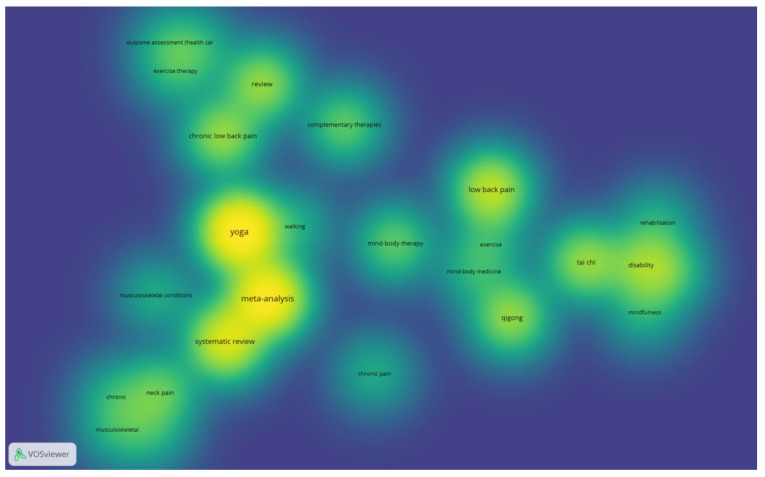
Density Visualization.

The network and density visualization analyses found some interrelated keywords (yoga, meta-analysis, and systematic reviews), which were the keywords often used in the included reviews (Figure 2 and Figure 3).

### 3.2. Overlapping

A total of 133 original trials were recovered in the included reviews. Of these, there were 48 trials without double counting. The overlap was very high between the trials for qigong (CCA = 36%), tai chi (CCA = 25%), and yoga (CCA = 16%). Supplementary File S5 shows all the citation matrices and the CCA calculations.

### 3.3. AMSTAR 2 Rating

Six reviews were rated as low quality [28,30,33,37,44,48] and fourteen were judged as critically low quality (Table 1).

### 3.4. Qigong for Chronic Spinal Pain

Table 2 shows the main characteristics and effect sizes of the six reviews included in this section [13,29,30,31,32,49].

#### 3.4.1. Qigong and Chronic Low Back Pain

The effects of qigong on chronic pain were inconsistent [13,31,32]. Only one review reported beneficial effects of pain reduction [32].

#### 3.4.2. Qigong and Chronic Neck Pain

Pain [29,30,49] and the physical component of quality of life [30] were meta-analyzed. Qigong improved quality of life after 12 weeks of intervention, but this effect was not maintained [30]. Meta-analyses often found that qigong was superior to waitlist in reducing chronic pain [29,30,49], but this effectiveness was not statistically significant compared to exercise [49].

### 3.5. Tai Chi for Chronic Spinal Pain

Table 3 shows the main characteristics and effect sizes of the five reviews included in this section [13,31,33,44,50]. No reviews were found on the effects of tai chi on chronic neck pain.

#### Tai Chi and Chronic Low Back Pain

Tai chi was found to be more effective than multiple controls in reducing chronic pain in most reviews [13,33,44,50]. One review did not show differences between groups [31].

### 3.6. Yoga for Chronic Spinal Pain

Table 4 shows the main characteristics and the effect sizes of the 13 reviews included in this section [13,28,31,34,35,36,37,45,46,47,48,51,52].

#### 3.6.1. Yoga and Chronic Low Back Pain

Chronic low back pain was the most common outcome of interest [13,28,31,34,35,36,37,45,47,48,51], and was often reduced when different yoga styles were applied. Yoga was not superior to education or usual care in reducing depression [48]. Yoga was also not superior to multiple controls in improving overall quality of life [51]. There were inconsistencies between some meta-analyses when yoga was used to modulate both components of quality of life: physical functioning and mental health [34,37,48].

#### 3.6.2. Yoga and Chronic Neck Pain

Yoga was more effective than multiple controls in reducing overall mood states [46,52]. Furthermore, this mindful exercise decreased mood states when they were meta-analyzed separately in anxiety or depression [46]. Positively, some meta-analyses also found that yoga showed more benefits in improving overall quality of life compared to exercise or usual care [46,52]. However, the effectiveness of yoga on chronic neck pain was inconclusive [46,52].

## 4. Discussion

This umbrella review aimed to summarize all available evidence on the effectiveness of qigong, tai chi, and yoga in people with chronic spinal pain and neck pain on psychological factors and quality of life. The effects of qigong on chronic low back and neck pain were inconsistent but showed positive effects in improving the physical component of quality of life in people with chronic neck pain 12 weeks after intervention. Tai chi was superior to controls in reducing chronic low back pain, but we did not find any systematic reviews that satisfied our criteria for chronic neck pain. Yoga was superior to multiple controls in reducing chronic low back pain, but its effects were inconsistent in chronic neck pain. In people with chronic neck pain, yoga improved the overall quality of life and was effective in reducing general mood states, anxiety, and depression, but did not improve the overall quality of life in people with chronic low back pain.

Qigong often showed inconsistent results across our umbrella review. Although some meta-analyses often found that qigong was superior to waitlist in reducing pain [29,30,49], this effect was not maintained when exercise was used as a control group. We speculate that inconsistencies could be related to how body movements develop. Qigong exercises are based on movements that are much simpler than tai chi or yoga, which could imply less motor variability and complexity [55]. For example, Baduanjin, one of the most common therapeutic forms of qigong and a traditional Chinese mind–body aerobic exercise of moderate intensity [56], is characterized by simple, slow, and relaxing movements. This exercise is easy to learn and has fewer physical and cognitive demands because it only contains eight simple movements, in contrast to tai chi and yoga [57,58]. We found that tai chi and yoga seemed to produce benefits in improving pain in people with chronic low back pain, but in chronic neck pain, these effects were inconsistent for yoga and no systematic reviews satisfied our criteria for tai chi and this condition. In this sense, new research comparing the effects among qigong, tai chi, and yoga could help us to understand whether the results found in this umbrella review were related to the difficulties of applied exercises or if mindful exercises could be a first line of treatment for reducing pain in some chronic spinal pain conditions. A recent recommendation for the management of chronic low back pain [59] suggested that exercise training interventions for this condition should include trunk-muscle strengthening and endurance, multimodal exercise interventions, specific trunk-muscle activation exercises, aerobic exercise, aquatic exercise, general exercise (Grade A), and movement-control exercise or trunk-mobility exercise (Grade B). In chronic neck pain, interventions such as reassurance, advice, education, physical activity, and exercise were recently recommended [60]. The reason why we did not observe relevant improvements regarding qigong in our umbrella review was possibly associated with the physical load and variability of this mindful exercise being inadequate to induce the necessary physical adaptations to improve chronic spinal pain conditions.

Yoga was found to improve quality of life and different psychological states (e.g., anxiety and depression) in people with chronic neck pain, but surprisingly, these positive effects were not maintained in populations with chronic low back pain. According to our findings, a large number of systematic reviews with a meta-analysis found inconsistent results regarding the aforementioned outcomes [61,62,63,64,65]. For example, systematic reviews showed that yoga can be an effective approach to reduce depressive disorders [62] and anxiety symptoms in some populations [61]. On the other hand, yoga often seemed to not produce significant effects on quality of life in different chronic diseases such as multiple sclerosis [63] or osteoarthritis [64]. Interestingly, yoga was found to improve quality of life in women with breast cancer, but this effect was not maintained when physical activities were included as control groups [65]. Therefore, readers should be aware that more research on the effectiveness of yoga in people with chronic disease is required to establish firm conclusions about the relevance of this intervention in people with chronic symptoms.

### 4.1. Clinical Implications

This umbrella review offered evidence to encourage health professionals to apply both tai chi and yoga when trying to improve pain in people with chronic spinal pain, especially in chronic low back pain. Above all, most reviews evaluated the effectiveness of yoga, which appears to be a promising approach to reduce not only chronic low back pain, but also other relevant outcomes (e.g., anxiety and depression) in chronic neck pain. Different yoga styles were often explored in the research, and some authors stated that there was no evidence that one style was better than another [66,67]. However, Hatta, Iyengar, or Viniyoga styles were used mainly in clinical research. These styles are based on adapting asanas to the requirements of everyone, strongly emphasizing how people align each asana during yoga practice. Therefore, we wondered if the therapeutic effects of yoga could be better when asanas, alignments, and doses were adapted and adjusted for everyone, such as how exercise therapy and motor control exercises are administered. Although asana is now often seen as the main component of yoga in Western society, musculoskeletal clinicians and clinical researchers should not forget to incorporate meditative, breathing (pranayama), and lifestyle strategies during yoga practice, which are at least as important as physical dimension [14,67,68] in achieving both physical and psychological well-being.

Unfortunately, musculoskeletal clinicians should be aware that important questions remain unresolved. First, we do not know what style of qigong, tai chi, or yoga could produce better results in this population. Second, we detected that some included reviews reported on clinical trials that did not only evaluate qigong, tai chi, or yoga as an isolated intervention. These trials mixed a large list of interventions under the umbrella of qigong, tai chi, or yoga programs [69,70,71,72,73]. Therefore, we encourage readers to be aware that some conclusions could be based on multidisciplinary interventions, and therefore, they should interpret the findings of this umbrella review with caution. Finally, we do not know whether all the clinical trials reported enough information to replicate. The TIDieR checklist [74] is a useful tool for detecting whether a clinical trial provided enough details to replicate its intervention in any environment (research or clinic). However, only one included review [50] used this tool; therefore, we do not know with certainty how replicable these qigong, tai chi, and yoga trials would be.

### 4.2. Methodological Considerations

The AMSTAR 2 tool often judged the methodological quality of the included reviews as critically low. This judgment emerged mainly because three critical domains were totally or partially unsatisfied (items 2, 7, and 11). Developing a review protocol should be mandatory to promote transparency and reduce potential biases [42]. However, many included reviews did not prospectively register their protocol, were incomplete, or did not provide justifications in terms of possible deviations. The study selection process is another important point to consider. Review authors should be transparent about how they carry out all their methodological processes. A list of excluded references in their last screening before selecting their included studies should be submitted at least as a supplementary file. Unfortunately, some included reviews did not provide this information. Methodological concerns also became visible in terms of how some meta-analyses were developed and how heterogeneity could impact on the findings of each meta-analysis. Surprisingly, the AMSTAR 2 tool did not consider whether a systematic review applied the GRADE approach or not. The GRADE framework is essential to know the certainty of each outcome that is evaluated in a systematic review. Unfortunately, this approach was only applied in 30% of the included reviews. Another important point is related to overlap. A clear objective of an umbrella review is to detect whether there is overlap between included reviews. This umbrella review found a very high overlap between the qigong, tai chi, and yoga trials. In this sense, readers should be aware that the conclusions of this umbrella review could be contaminated by these overlaps. Finally, some recent umbrella reviews [75,76] assessed the certainty of evidence using the *2018 Physical Activity Guidelines Advisory Committee Scientific Report* [77] and calculated a meta-meta-analysis, a new generation of meta-analysis [78]. However, we did not develop any of them for one critical reason: the presence of a very high overlap between the included reviews. We believed that we needed to be cautious before combining the findings of different reviews that included the same clinical trials, which could have underestimated or overestimated our findings. Readers should take this into account. All these results should help review authors and editors to reflect on the need to develop and/or publish more systematic reviews covering the same topics.

### 4.3. Future Agenda

Some important gaps were found in the literature on the potential effectiveness of qigong, tai chi, and yoga in people with chronic spinal pain. When possible, future systematic reviews could aim to: (i) incorporate the GRADE approach; (ii) apply the TIDieR checklist; (iii) evaluate whether tai chi is an effective approach to improve outcomes in chronic neck pain; and (iv) meta-analyze psychological factors that have been shown to be important between people with chronic spinal pain such as fear related to pain [79] or pain catastrophizing [80].

### 4.4. Limitations

The results of this umbrella review were based on how other review authors analyzed and interpreted original research trials. We know that many trials that were included not only evaluated qigong, tai chi, or yoga, but also sometimes mixed these approaches with other interventions. We encourage readers to interpret the findings of this umbrella review with caution. Only publications in English and Spanish were considered, and theses and conference abstracts were not included. In this sense, some important information could be missed. The certainty of the evidence and meta-meta-analyses regarding the effectiveness of qigong, tai chi, and yoga in modulating chronic spinal outcomes were not calculated due to the presence of a very high overlap between the included reviews. The ROBIS tool [81] is another interesting instrument to assess the risk of bias of systematic reviews. We did not use the ROBIS tool, and recent evidence supports that AMSTAR 2 and the ROBIS tool address a large number of same or similar constructs [82]. However, we recognize that some critical items of the ROBIS tool were not covered by the AMSTAR 2 tool (e.g., restrictions within eligibility criteria or completeness of data extracted for analyses) [82].

## 5. Conclusions

This umbrella review concluded that:

1. The effect of qigong on chronic back and neck pain was often inconsistent.
2. Qigong seemed to be effective in improving the physical component of quality of life only 12 weeks after the intervention.
3. Tai chi could be an interesting approach to reduce chronic low back pain.
4. No meta-analyses satisfied our criteria regarding tai chi and outcomes of interest in chronic neck pain.
5. Yoga could improve chronic low back pain.
6. A lack of relevant effects was found for yoga in reducing depression and improving overall quality of life in chronic low back pain.
7. The effects of yoga on both components of quality of life (mental and physical) were inconsistent in chronic low back pain.
8. Yoga could be an effective approach to decreasing anxiety, depression, and overall mood states and improving overall quality of life in chronic neck pain.
9. Inconsistencies were found that were associated with the effects of yoga on chronic neck pain.
10. More well-designed research is required on our covered topic to solve the clinical and methodological concerns that were discussed in this umbrella review.

## Figures and Tables

**Table 1:** The AMSTAR 2 tool.

Author(s)	1	2	3	4	5	6	7	8	9	10	11	12	13	14	15	16	Overall Score
Anheyer et al., 2021 [34]																	CLQR
Bai et al., 2015 [29]																	CLQR
Cramer et al., 2013 [51]																	CLQR
Cramer et al., 2017 [52]																	CLQR
Gross et al., 2015 [30]																	LQR
Hall et al., 2017 [50]																	CLOR
Holtzman et al., 2013 [45]																	CLQR
Kim 2020 [35]																	CLQR
Kong et al., 2016 [44]																	LQR
Li et al., 2019 [46]																	CLQR
Nduwimana et al., 2020 [31]																	CLQR
Qin et al., 2019 [33]																	LQR
Skelly et al., 2020 [28]																	LQR
Slade et al., 2007 [36]																	CLQR
Ward et al., 2013 [47]																	CLQR
Wieland et al., 2017 [48]																	LQR
Yuan et al., 2015 [49]																	CLQR
Zhang et al., 2019 [32]																	CLQR
Zhu et al., 2020 [37]																	LQR
Zou et al., 2019 [13]																	CLQR

**Answers:** Red color: No; yellow colour: Partially yes; green colour: Yes. Overall Score: CLQR: Critically Low. Quality Review LQR: Low-Quality Review. **Items: AMSTAR 1:** Did the research questions and inclusion criteria for the review include the components of PICO? **AMSTAR 2:** Did the report of the review contain an explicit statement that the review methods were established prior to the conduct of the review and did the report justify any significant deviations from the protocol? **AMSTAR 3:** Did the review authors explain their selection of the study designs for inclusion in the review? **AMSTAR 4:** Did the review authors use a comprehensive literature search strategy? **AMSTAR 5:** Did the review authors perform study selection in duplicate? **AMSTAR 6:** Did the review authors perform data extraction in duplicate? **AMSTAR 7:** Did the review authors provide a list of excluded studies and justify the exclusions? **AMSTAR 8:** Did the review authors describe the included studies in adequate detail? **AMSTAR 9:** Did the review authors use a satisfactory technique for assessing the risk of bias in individual studies that were included in the review? **AMSTAR 10:** Did the review authors report on the sources of funding for the studies included in the review? **AMSTAR 11:** If a meta-analysis was performed, did the review authors use appropriate methods for statistical combination of results? **AMSTAR 12:** If a meta-analysis was performed, did the review authors assess the potential impact of the risk of bias in individual studies on the results of the meta-analysis or other evidence syntheses? **AMSTAR 13:** Did the review authors account for the risk of bias in individual studies when interpreting/discussing the results of the review? **AMSTAR 14:** Did the review authors provide a satisfactory explanation for and discussion of any heterogeneity observed in the results of the review? **AMSTAR 15:** If they performed a quantitative synthesis, did the review authors carry out an adequate investigation of publication bias (small-study bias) and discuss its likely impact on the results of the review? **AMSTAR 16:** Did the review authors report any potential sources of conflict of interest, including any funding they received for conducting the review?

**Table 2:** Included reviews: qigong.

Study and Year	Quality Assessment	RCTs Included in This Umbrella	Participants	Interventions	Outcome Measurements	Effect Sizes
CHRONIC LOW BACK PAIN						
Nduwimana et al., 2020 [31]	GRADE Unavailable Tool for quality assessment The PEDro scale	3	375 with chronic low back pain	EXPERIMENTAL Qigong CONTROL Exercise, or no intervention, or waitlist	Short-term (ST) effects: 0–3 months after the intervention Intermediate-term (IT) effects: 3–6 months postintervention	SMD (95% CI): subgroup analysis according to the type of intervention and the outcome measurement time points 1. Pain—qigong vs. exercise, no intervention, and waitlist: a. ST effect: −1.34 (−3.19 to 0.51), *p* = 0.16; I^2^ = 98% b. IT effect: 0.12 (−2.67 to 2.91), *p* = 0.93; I^2^ = 99%
Zhang et al., 2019 [32]	GRADE Unavailable Tool for quality assessment The PEDro scale	3	375 with chronic low back pain	EXPERIMENTAL Qigong CONTROL Exercise or waitlist	Unspecified	Hedge’s g (95% CI): subgroup analysis for the type of experimental group 1. Pain—qigong vs. exercise and waitlist: −0.54 (−0.86 to −0.23), *p* < 0.001; I^2^ = 75.9%
Zou et al., 2019 [13]	GRADE Unavailable Tool for quality assessment The PEDro scale	2	303 with chronic low back pain	EXPERIMENTAL Qigong CONTROL Exercise or waitlist	Authors declared that none of included studies used follow-up assessments	SMD (95% CI): subgroup analysis according to the type of experimental group 1. Pain—qigong vs. exercise and waitlist: −0.21 (−0.48 to 0.06), *p* = 0.12; I^2^ = 10.0%
CHRONIC NECK PAIN						
Bai et al., 2015 [29]	GRADE Unavailable Tool for quality assessment The Cochrane risk of bias tool	2	240 with chronic neck pain	EXPERIMENTAL Internal qigong CONTROL Waitlist	3-month follow-up 6-month follow-up	SMD (95% CI): subgroup analysis according to clinical condition and the outcome measurement time points 1. Pain—internal qigong vs. waitlist: a. At 3 months: −1.17 (−2.44 to 0.10), *p* = 0.07; I^2^ = 93% b. At 6 months: −1.00 (−1.94 to −0.06), *p* = 0.04; I^2^ = 87%
Gross et al., 2015 [30]	GRADE Available Tool for quality assessment The Cochrane risk of bias tool	2	240 with chronic neck pain	EXPERIMENTAL Internal qigong CONTROL Waitlist	12 weeks of treatment 24 weeks of treatment	MD (95% CI): subgroup analysis according to the type of intervention and the outcome measurement time points 1. Pain—internal qigong vs. waitlist: a. 12 weeks of treatment: −13.28 (−20.98 to −5.58), *p* = 0.00073; I^2^ = 0% b. 24 weeks of treatment: −7.82 (−14.57 to −1.07), *p* = 0.023; I^2^ = 0% 2. Quality of life (physical component)—internal qigong vs. waitlist: a. 12 weeks of treatment: −2.72 (−5.42 to −0.01), *p* = 0.049; I^2^ = 0% b. 24 weeks of treatment: −1.88 (−5.80 to 2.04), *p* = 0.35; I^2^ = 45%
Yuan et al., 2015 [49]	GRADE Available Tool for quality assessment The Cochrane risk of bias tool	2	240 with chronic neck pain	EXPERIMENTAL Internal qigong CONTROL Exercise or waitlist	ST: <3 months IT: ~3–12 months	WMD (95% CI): subgroup analysis according to the type of control group and the outcome measurement time points: 1. Pain—internal qigong vs. waitlist; *p*-value vas not reported: a. ST effect: −15.27 (−22.49 to −8.05); I^2^ = 47.5% b. IT effect: −10.18 (−16.63 to −3.73); I^2^ = 0% 2. Pain—internal qigong vs. exercise: a. ST effect: 1.88 (−5.77 to 9.54), *p* = 0.63; I^2^ = 0% b. IT effect: 1.00 (−6.21 to 8.21), *p* = 0.79; I^2^ = 0%

CI = confidence interval; GRADE = Grading of Recommendations, Assessment, Development and Evaluation; MD = mean difference; RCTs = randomized controlled trials; SMD = standardized mean difference; WMD = weighted mean difference. Blue color: statistically significant results observed.

**Table 3:** Included reviews: tai chi.

Study and Year	Quality Assessment	RCTs Included in This Umbrella	Participants	Interventions	Outcome Measurements	Effect Sizes
CHRONIC LOW BACK PAIN						
Hall et al., 2017 [50]	GRADE Available Tool for quality assessment The Cochrane risk of bias tool	2	349 with chronic low back pain	EXPERIMENTAL Tai chi CONTROL Attention control, no, usual care, or waitlist	Unspecified	SMD (95%): subgroup analysis according to clinical condition 1. Pain—tai chi vs. attention control, usual care, or waitlist: −0.84 (−1.27 to −0.42), *p* < 0.0001; I^2^ = 69%
Kong et al., 2016 [44]	GRADE Unavailable Tool for quality assessment The PEDro scale	3	385 with chronic low back pain	EXPERIMENTAL Tai chi CONTROL Physical therapy or waitlist plus health care	Immediately after the treatments—up to 1 day	SMD (95%): subgroup analysis according to clinical condition 1. Pain—tai chi vs. physical therapy or waitlist plus health care: −0.81 (−1.11 to −0.52), *p* < 0.00001; I^2^ = 46%
Nduwimana et al., 2020 [31]	GRADE Unavailable Tool for quality assessment The PEDro scale	2	480 with chronic low back pain	EXPERIMENTAL Tai chi CONTROL Exercise (swimming, jogging), no exercise, usual care, or waitlist	Short-term (ST) effects: 0–3 months after the intervention	SMD (95%CI): subgroup analysis according to the type of experimental group and outcome measurement time points: 1. ST pain—tai chi vs. exercise, no exercise, usual care, and waitlist: −1.19 (−2.97 to 0.58), *p* = 0.19; I^2^ = 99%
Qin et al., 2019 [33]	GRADE Unavailable Tool for quality assessment The PEDro scale	3	252 with chronic low back pain	EXPERIMENTAL Tai chi CONTROL No intervention or waitlist	Unspecified	MD (95%): subgroup analysis according to the type of control group 1. Pain—tai chi vs. no intervention, usual care, or waitlist −1.71 (−2.31 to −1.11), *p* < 0.00001; I2 = 82%
Zou et al., 2019 [13]	GRADE Unavailable Tool for quality assessment The PEDro scale	2	203 with chronic low back pain	EXPERIMENTAL Tai chi CONTROL Exercise, no intervention, or waitlist	Authors declared that none of included studies used follow-up assessments	SMD (95%): subgroup analysis according to the type of experimental group 1. Pain—tai chi vs. exercise, no intervention, or waitlist: −0.75 (−1.05 to −0.46), *p* < 0.001; I^2^ = 0%

CI = confidence interval; GRADE = Grading of Recommendations, Assessment, Development and Evaluation; MD = mean difference; RCTs = randomized controlled trials; SMD = standardized mean difference; ST = short-term. Blue color: statistically significant results observed.

**Table 4:** Included reviews: yoga.

Study and Year	Quality Assessment	RCTs Included in This Umbrella	Participants	Interventions	Outcome Measurements	Effect Sizes
CHRONIC LOW BACK PAIN						
Anheyer et al., 2021 [34]	GRADE Unavailable Tool for quality assessment The Cochrane risk of bias tool	19	2250 with chronic low back pain	EXPERIMENTAL Yoga (Kundalini, Iyengar, Hatha, Vinyasa, therapeutic approach, or integrated approach) with or without usual care CONTROL Exercise, lifestyle advice, multicomponent intervention, usual care, or waitlist	Short-term (ST) effects: postintervention and closest to 12 weeks after randomization Long-term (LT) effects: closest to 6 months after randomization	SMD (95% CI): subgroup analysis according to outcome measurement time points—*p* value was not reported. 1. Pain—yoga vs. passive control (usual care and/or waitlist) a. LT (6 months and longer): −0.29 (−0.47 to −0.11); I^2^ = 33% 2. Pain—yoga vs. active control: a. LT (6 months and longer): −0.31 (−1.55 to 0.93); I^2^ = 91% 3. Quality of life (physical component)—yoga vs. passive control (usual care and/or waitlist): a. ST (2 to 4 months): 0.28 (0.10 to 0.47); I^2^ = 24% b. LT (6 months and longer): 0.22 (0.03 to 0.41); I^2^ = 0% 4. Quality of life (physical component)—yoga vs. active control: a. ST (2 to 4 months): 0.51 (−0.03 to 1.05); I^2^ = 88% b. LT (6 months and longer): 0.31 (−1.95 to 2.56); I^2^ = 93% 5. Quality of life (mental component)—yoga vs. passive control (usual care and/or waitlist): a. ST (2 to 4 months): 0.17 (0.02 to 0.32); I^2^ = 0% b. LT (6 months and longer): 0.13 (−0.23 to 0.48); I^2^ = 39% 6. Quality of life (mental component)—yoga vs. active control: a. ST (2 to 4 months): 0.57 (−0.25 to 1.40); I^2^ = 92% b. LT (6 months and longer): 0.64 (−7.81 to 9.10); I^2^ = 93%
Cramer et al., 2013 [51]	GRADE Unavailable Tool for quality assessment The Cochrane risk of bias tool	8	832 with chronic low back pain	EXPERIMENTAL Yoga (Iyengar, Hatha, Viniyoga), with or without education, usual care, or vegetarian diet CONTROL Education, exercise, multicomponent intervention, usual care, or waitlist	ST: after the end of the intervention and closest to 12 weeks after randomization LT: closest to 12 months after randomization	SMD (95% CI): subgroup analysis according to outcome measurement time points 1. Pain—yoga vs. active and passive controls: a. ST: −0.48 (−0.65 to −0.31), *p* < 0.00001; I^2^ = 0% b. LT: −0.33 (−0.59 to −0.07), *p* = 0.01; I^2^ = 48% 2. General quality of life—yoga vs. active and passive controls: a. ST: 0.41 (−0.11 to 0.93), *p* = 0.12; I^2^ = 72% b. LT: 0.18 (−0.05 to 0.41), *p* = 0.13; I^2^ = 0%
Holtzman et al., 2013 [45]	GRADE Unavailable Tool for quality assessment CLEAR NPT	6	522 with chronic low back pain	EXPERIMENTAL Yoga (Hatha, Viniyoga, Iyengar) CONTROL Exercise, education, or waitlist	Post-treatment analysis: the earliest assessment of the outcome variables after treatment Follow-up analysis: the assessment closest to three months postintervention	Cohen’s d (95%): subgroup analysis according to outcome measurement time points *—p* value was not reported 1. Pain—yoga vs. exercise, education, or waitlist: a. Post-treatment analysis: 0.623 (0.377 to 0.868); I^2^ = 22.4% b. Follow-up analysis: 0.397 (0.053 to 0.848); I^2^ = 74.8%
Kim 2020 [35]	GRADE Unavailable Tool for quality assessment The Cochrane risk of bias tool	6	523 with chronic low back pain	EXPERIMENTAL Yoga (Iyengar, Hatha, Viniyoga) CONTROL Education, usual care, or waitlist	After 12 weeks of treatment	SMD (95%): overall effect 1. Pain—yoga vs. education, usual care, or waitlist: −0.41 (−0.58 to −0.23), *p* < 0.0001; I^2^ = 0%
Nduwimana et al., 2020 [31]	GRADE Unavailable Tool for quality assessment The PEDro scale	4	241 with chronic low back pain	EXPERIMENTAL Yoga CONTROL Unspecified	Intermediate-term (IT) effects: 3–6 months postintervention	SMD (95%CI): subgroup analysis according to the type of experimental group and outcome measurement time points: 1. Pain—yoga vs. control group: a. IT: −1.70 (−3.52 to 0.12), *p* = 0.07; I^2^ = 97%
Skelly et al., 2020 [28]	GRADE Available Tool for quality assessment The Cochrane risk of bias tool	9	1221 with chronic low back pain	EXPERIMENTAL Yoga (Hatha, Kundalini, Iyengar, Viniyoga) CONTROL Attention control, exercise, usual care, or waitlist	ST: 1 to <6 months following treatment completion IT: >6 to <12 months	MD (95%): subgroup analysis according to the type of control group and outcome measurement time points: 1. Pain—yoga vs. attention control or waitlist: a. ST: −0.87 (−1.49 vs. −0.24) *p* = 0.014; I^2^ = 64.1% b. IT: −1.16 (−2.16 to −0.27), *p* = 0.029; I^2^ = 0% 2. Pain—yoga vs. exercise: a. ST: −0.63 (−1.68 to 0.45), *p* = 0.196; I^2^ = 87.5%
Slade et al., 2007 [36]	GRADE Unavailable Tool for quality assessment The PEDro scale	2	145 with chronic low back pain	EXPERIMENTAL Yoga (Viniyoga, Iyengar) CONTROL No exercises plus education	IT: 26 to 32 weeks	SMD (95%): subgroup analysis outcome measurement time points—*p* value and heterogeneity (I^2^) were not reported 1. Pain—yoga vs. education or no exercise: a. IT: 0.92 (0.47 to 1.37)
Ward et al., 2013 [47]	GRADE Unavailable Tool for quality assessment The Cochrane risk of bias tool and The PEDro scale	4	449 with chronic low back pain	EXPERIMENTAL Yoga (Hatha, Viniyoga, Iyengar) CONTROL Exercise, usual care, or waitlist	Unspecified	SMD (95%): overall effect: 1. Pain—yoga vs. exercise, usual care, or waitlist: −0.61 (−0.97 to −0.26), *p* = 0.0007; I^2^ = 63%
Wieland et al., 2017 [48]	GRADE Available Tool for quality assessment The Cochrane risk of bias tool and	6	565 with chronic low back pain	EXPERIMENTAL Yoga (Iyengar, Hatha) CONTROL Education or usual care	ST: 4 to 6 weeks SIT: 3 to 4 months IT: 6 months LT: 12 months	MD (95%CI): subgroup analysis according to outcome measurement time points: 1. Pain—Yoga vs. education or usual care: a. ST: −10.83 (−20.85 to −0.81), *p* = 0.034: I^2^ = 0% b. SIT (3 to 4 months): −4.55 (−7.04 to −2.06), *p* = 0.00035; I^2^ = 0% c. IT: −7.81 (−13.37 to −2.25), *p* = 0.0059; I^2^ = 64% d. LT: −5.40 (−14.50 to 3.70), *p* = 0.24; I^2^ = 79% SMD (95%CI): subgroup analysis according to outcome measurement time points: 2. Quality of life (physical component)—yoga vs. education or usual care: a. SIT: 0.22 (0.00 to 0.44), *p* = 0.051; I^2^ = 0% 3. Quality of life (mental component)—yoga vs. education or usual care: a. SIT: 0.20 (−0.02 to 0.41), *p* = 0.081; I^2^ = 0% 4. Depression—yoga vs. education or usual care: a. SIT (3 months): −0.15 (−0.49 to 0.19), *p* = 0.39; I^2^ = 0%
Zhu et al., 2020 [37]	GRADE Available Tool for quality assessment The Cochrane risk of bias tool and	17	1921 with chronic low back pain	EXPERIMENTAL Yoga (Hatha, Iyengar, Viniyoga) CONTROL Education, exercise, no treatment, physical therapy, or usual care	ST, IT, and LT differed in different subgroup analyses	MD (95%CI): subgroup analysis according to the type of control group and outcome measurement time points: 1. Pain—yoga vs. non-exercise: a. ST (4 to 8 weeks): −0.83 (−1.19 to −0.48), *p* < 0.00001; I^2^ = 0% b. SIT (3 months): −0.43 (−0.64 to −0.23), *p* < 0.0001; I^2^ = 0% c. IT (6 to 7 months): −0.56 (−1.02 to −0.11), *p* = 0.02; I^2^ = 50% d. LT (12 months): −0.52 (−1.64 to 0.59), *p* = 0.36; I^2^ = 87% 2. Pain—yoga vs. physical therapy exercise: a. ST (7 days of intensive intervention): −2.36 (−3.15 to −1.56), *p* < 0.00001; I^2^ = 0% b. ST (4 to 10 weeks): −0.37 (−1.16 to 0.42), *p* = 0.36; I^2^ = 81% c. SIT (3 months): 0.19 (−0.63 to 1.01), *p* = 0.65; I^2^ = 64% d. IT (6 months): −0.73 (−2.13 to 0.67), *p* = 0.31; I^2^ = 85% 3. Quality of life (physical component)—yoga vs. physical therapy exercise: a. SIT (3 months): 0.18 (−1.97 to 2.32), *p* = 0.87; I^2^ = 0% 4. Quality of life (mental component)—yoga vs. physical therapy exercise: a. SIT (3 months): 0.07 (−2.74 to 2.89), *p* = 0.96; I^2^ = 0% Results were from a subgroup analysis according to the type of control group and outcome measurement time points: SMD (95%CI) 5. Quality of life (physical component)—yoga vs. non-exercise: a. SIT (3 months): 0.06 (−0.10 to 0.22), *p* = 0.48; I^2^ = 0% b. IT (6 months): 0.08 (−0.13 to 0.28), *p* = 0.45; I^2^ = 0% 6. Quality of life (mental component)—yoga vs. non-exercise: a. SIT (3 months): 0.15 (−0.01 to 0.31), *p* = 0.06; I^2^ = 0% b. IT (6 months): 0.18 (−0.03 to 0.39), *p* = 0.09; I^2^ = 0%
Zou et al., 2019 [13]	GRADE Unavailable Tool for quality assessment The PEDro scale	8	1237 with chronic low back pain	EXPERIMENTAL Yoga (group and home practice) CONTROL Education, exercise, self-care, or waitlist	Authors declared that none of studies used follow-up assessments	SMD (95%): subgroup analysis according to the type of experimental group: 1. Pain—yoga vs. education, exercise, self-care, or waitlist: −0.33 (−0.47 to −0.19), *p* = 0.001; I^2^ = 33.7%
CHRONIC NECK PAIN						
Cramer et al., 2017 [52]	GRADE Unavailable Tool for quality assessment The Cochrane risk of bias tool		188 with chronic neck pain	EXPERIMENTAL Yoga (Iyengar) with physiotherapy CONTROL Usual care	ST was not defined	SMD (95%): overall effects 1. Pain—yoga vs. usual care: −1.28 (−1.81 to −0.75), *p* < 0.00001; I^2^ = 62% 2. General quality of life—yoga vs. usual care: 0.57 (0.17 to 0.97), *p* = 0.006; I^2^ = 20% 3. Mood—yoga vs. usual care: −1.02 (−1.38 to −0.65), *p* < 0.00001; I^2^ = 0%

CI = confidence interval; CLEAR NPT = checklist to evaluate a report of a nonpharmacological trial; GRADE = Grading of Recommendations, Assessment, Development and Evaluation; IT = intermediate-term; LT = long-term; MD = mean difference; RCTs = randomized controlled trials; RD = risk difference; SIT = short-intermediate-term; SMD = standardized mean difference; ST = short-term. Blue color: statistically significant results observed.

## Data Availability

Not applicable.

## Supplementary Materials

The following supporting information can be downloaded at: https://www.mdpi.com/article/10.3390/ijerph191912062/s1, Supplementary File S1. Search strategies, Supplementary File S2. List of excluded studies, Supplementary File S3. Reasons for exclusions, Supplementary File S4. Reviews evaluated after manual searches, Supplementary File S5. Citation matrices and CCA.
