# Supplementary material for: The Effects of Mind–Body Exercises on Chronic Spinal Pain Outcomes: A Synthesis Based on 72 Meta-Analyses
Source: Int J Environ Res Public Health. 2022 Sep 23;19(19):12062. doi: 10.3390/ijerph191912062 (PMC9564899; doi:10.3390/ijerph191912062)
Supplement: Supplementary file 1 [file ijerph-19-12062-s001.zip › Supplementary File S1.pdf]

Supplementary File S1. Search Strategies**.**

CINAHL (date 06/03/2022)

TX (neck OR whiplash OR cervical OR low back OR thoracic OR spinal OR spine) AND TX (qigong OR qi gong OR Tai OR T'ai OR Yoga OR chi kung OR Baduanjin OR Wuqinxi OR Liuzijue OR Yijinjing)

(studies retrieved = 979)

Filters: Type of publication: Meta Analysis, Meta Synthesis, Systematic Review

Magnifiers - Search also within the full text of articles

Search modes - Boolean/Phrase

Cochrane Library (date 06/03/2022)

#1 (qigong) en Revisiones Cochrane

#2 (qi gong) en Revisiones Cochrane

#3 (Tai) en Revisiones Cochrane

#4 (T'ai) en Revisiones Cochrane

#5 (Yoga) en Revisiones Cochrane

#6 (chi kung) en Revisiones Cochrane

#7 (Baduanjin) en Revisiones Cochrane

#8 (Wuqinxi) en Revisiones Cochrane

#9 (Liuzijue) en Revisiones Cochrane

#10 (Yijinjing) en Revisiones Cochrane

#11 (cervical) en Revisiones Cochrane

#12 (neck) en Revisiones Cochrane

#13 (whiplash) en Revisiones Cochrane

#14 (low back) en Revisiones Cochrane

#15 (thoracic) en Revisiones Cochrane

#16 (spinal) en Revisiones Cochrane

#17 (spine) en Revisiones Cochrane

#18 ((#1 OR #2 OR #3 OR #4 OR #5 OR #6 OR #7 OR #8 OR #9 OR #10) AND (#11 OR #12 OR #13 OR #14 OR #15 OR #16 OR #17)) en Revisiones Cochrane

(Cochrane reviews retrieved = 200)

Embase (date 06/03/2022)

('neck'/exp OR neck OR 'whiplash'/exp OR whiplash OR cervical OR 'low back' OR (low AND ('back'/exp OR back)) OR thoracic OR spinal OR 'spine'/exp OR spine) AND ('qigong'/exp OR qigong OR 'qi gong'/exp OR 'qi gong' OR (('qi'/exp OR qi) AND gong) OR tai OR t`ai OR 'yoga'/exp OR yoga OR 'chi kung'/exp OR 'chi kung' OR (chi AND kung) OR yijinjing OR 'baduanjin'/exp OR baduanjin OR wuqinxi OR liuzijue) AND ([systematic review]/lim OR [meta analysis]/lim)

(studies retrieved = 280)

Key words were searched in all fields.

PsycINFO (date 06/03/2022)

(neck OR whiplash OR cervical OR low back OR thoracic OR spinal OR spine) AND (qigong OR qi gong OR Tai OR T’ai OR Yoga OR chi kung OR Baduanjin OR Wuqinxi OR Liuzijue OR Yijinjing)

(studies retrieved = 18)

Filters: Limited by: Methodology: Meta-analysis, Meta-syntesis, Systematic Review

Key words were searched in all fields.

PubMed (date 06/03/2022)

(neck [all] OR whiplash [all] OR cervical [all] OR low back [all] OR thoracic [all] OR spinal [all] OR spine [all]) AND (qigong [all] OR qi gong [all] OR Tai Ji [all] OR Tai [all] OR T'ai [all] OR Yoga [all] OR chi kung [all] OR Baduanjin [all] OR Wuqinxi [all] OR Liuzijue [all] OR Yijinjing [all])

(studies retrieved = 109)

Filters: Systematic Review, Meta-Analysis

SPORTDiscus (date 06/03/2022)

TI (meta review OR systematic review OR meta-analysis) AND TX (qigong OR qi gong OR Tai OR T’ai OR Yoga OR chi kung OR Baduanjin OR Wuqinxi OR Liuzijue OR Yijinjing) AND TX (neck OR whiplash OR cervical OR low back OR thoracic Or spinal OR spine)

(studies retrieved = 252)
