# Supplementary material for: The Effects of Mind–Body Exercises on Chronic Spinal Pain Outcomes: A Synthesis Based on 72 Meta-Analyses
Source: Int J Environ Res Public Health. 2022 Sep 23;19(19):12062. doi: 10.3390/ijerph191912062 (PMC9564899; doi:10.3390/ijerph191912062)
Supplement: Supplementary file 1 [file ijerph-19-12062-s001.zip › Supplementary File S2.pdf]

Supplementary File S2. List of reviews that were excluded by specific reasons (n = 154).

	Review excluded	Reason
1.	Ahern MM, et al. The Effectiveness of Virtual Reality in Patients With Spinal Pain: A Systematic Review and Meta-Analysis. Pain Pract. 2020 Jul;20(6):656-675.	The intervention of interest was not meta-analyzed separately from other interventions.
2.	Ammendolia C, et al. Nonoperative treatment of lumbar spinal stenosis with neurogenic claudication: a systematic review. Spine (Phila Pa 1976). 2012 May 1;37(10):E609-16.	No intervention of interest.
3.	Ashe MC, et al. Physical Activity and Bone Health in Men: A Systematic Review and Meta-Analysis. J Bone Metab. 2021 Feb;28(1):27-39.	No chronic spinal pain.
4.	Baker MK, et al. Multi-modal exercise programs for older adults. Age Ageing. 2007 Jul;36(4):375-81.	No chronic spinal pain.
5.	Barros Dos Santos AO, et al. Effects of physical exercise on low back pain and cortisol levels: a systematic review with meta-analysis of randomized controlled trials. Pain Manag. 2021 Jan;11(1):49-57.	The intervention of interest was not meta-analyzed separately from other interventions.
6.	Benedetti MG, et al. The Effectiveness of Physical Exercise on Bone Density in Osteoporotic Patients. Biomed Res Int. 2018 Dec 23;2018:4840531.	No chronic spinal pain.
7.	Bettany-Saltikov J, et al. Surgical versus non-surgical interventions in people with adolescent idiopathic scoliosis. Cochrane Database Syst Rev. 2015 Apr 24;(4):CD010663.	No intervention of interest.
8.	Binder AI. Neck pain. BMJ Clin Evid. 2008 Aug 4;2008:1103.	The intervention of interest was not meta-analyzed.
9.	Bolam KA, et al. The effect of physical exercise on bone density in middle-aged and older men: a systematic review. Osteoporos Int. 2013 Nov;24(11):2749-62.	No chronic spinal pain.
10.	Braun J, et al. Treatment of ankylosing spondylitis and other spondyloarthritides. Curr Opin Rheumatol. 2009 Jul;21(4):324-34.	No chronic spinal pain.
11.	Brewer S, et al. Workplace interventions to prevent musculoskeletal and visual symptoms and disorders among computer users: a systematic review. J Occup Rehabil. 2006 Sep;16(3):325-58.	No intervention of interest.

	Review excluded	Reason
12.	Brinzo JA, et al. The effect of yoga on depression and pain in adult patients with chronic low back pain: a systematic review protocol. JBI Database System Rev Implement Rep. 2016 Jan;14(1):56-66.	Review protocol.
13.	Brox JI, et al. Systematic review of back schools, brief education, and fear-avoidance training for chronic low back pain. Spine J. 2008 Nov-Dec;8(6):948-58.	The intervention of interest was not meta-analyzed.
14.	Bussières AE, et al. The Treatment of Neck Pain-Associated Disorders and Whiplash-Associated Disorders: A Clinical Practice Guideline. J Manipulative Physiol Ther. 2016 Oct;39(8):523-564.e27.	The intervention of interest was not meta-analyzed.
15.	Büssing A, et al. Effects of yoga interventions on pain and pain-associated disability: a meta-analysis. J Pain. 2012 Jan;13(1):1-9.	Chronic spinal pain was not meta-analyzed separately from other conditions.
16.	Cai Z, et al. Effects of Tai Chi Chuan on Cognitive Function in Older Adults with Cognitive Impairment: A Systematic and Meta-Analytic Review. Evid Based Complement Alternat Med. 2020 Dec 28;2020:6683302.	No chronic spinal pain.
17.	Chang DG, et al. Yoga as a treatment for chronic low back pain: A systematic review of the literature. J Orthop Rheumatol. 2016 Jan 1;3(1):1-8.	The intervention of interest was not meta-analyzed.
18.	Choi BK, et al. Exercises for prevention of recurrences of low-back pain. Cochrane Database Syst Rev. 2010 Jan 20;2010(1):CD006555.	No intervention of interest.
19.	Chou R, et al. Nonpharmacologic therapies for acute and chronic low back pain: a review of the evidence for an American Pain Society/American College of Physicians clinical practice guideline. Ann Intern Med. 2007 Oct 2;147(7):492-504.	The intervention of interest was not meta-analyzed.
20.	Chou R, et al. Nonpharmacologic Therapies for Low Back Pain: A Systematic Review for an American College of Physicians Clinical Practice Guideline. Ann Intern Med. 2017 Apr 4;166(7):493-505.	The intervention of interest was not meta-analyzed.
21.	Chow TH, et al. The effect of Chinese martial arts Tai Chi Chuan on prevention of osteoporosis: A systematic review. J Orthop Translat. 2017 Jun 26;12:74-84.	No chronic spinal pain.

	Review excluded	Reason
22.	Collado-Mateo D, et al. Effect of exergames on musculoskeletal pain: A systematic review and meta-analysis. Scand J Med Sci Sports. 2018 Mar;28(3):760-771.	The intervention of interest was not meta-analyzed separately from other interventions.
23.	Côté P, et al. Management of neck pain and associated disorders: A clinical practice guideline from the Ontario Protocol for Traffic Injury Management (OPTIMa) Collaboration. Eur Spine J. 2016 Jul;25(7):2000-22.	The intervention of interest was not meta-analyzed.
24.	Cramer H, et al. Yoga for rheumatic diseases: a systematic review. Rheumatology (Oxford). 2013 Nov;52(11):2025-30.	The intervention of interest was not meta-analyzed.
25.	Crevelario De Melo R, et al. Effectiveness and safety of yoga to treat chronic and acute pain: a rapid review of systematic Reviews. BMJ Open. 2021; 11:e048536.	Overview of reviews.
26.	Crocker T, et al. Physical rehabilitation for older people in long-term care. Cochrane Database Syst Rev. 2013 Feb 28;(2):CD004294.	No chronic spinal pain.
27.	Crow EM, et al. Effectiveness of Iyengar yoga in treating spinal (back and neck) pain: A systematic review. Int J Yoga. 2015 Jan;8(1):3-14.	The intervention of interest was not meta-analyzed.
28.	Danielsson L, et al. Exercise in the treatment of major depression: a systematic review grading the quality of evidence. Physiother Theory Pract. 2013 Nov;29(8):573-85.	No chronic spinal pain.
29.	De Zoete RMJ, et al. The effectiveness of general physical exercise for individuals with chronic neck pain: a systematic review of randomised controlled Trials. European Journal of Physiotherapy. 2019;22(3):1-7.	The intervention of interest was not meta-analyzed.
30.	De Zoete RM, et al. Comparative effectiveness of physical exercise interventions for chronic non-specific neck pain: a systematic review with network meta-analysis of 40 randomised controlled trials. Br J Sports Med. 2020 Nov 2:bjsports-2020-102664.	Network meta-analysis.
31.	Denham-Jones L, et al. A systematic review of the effectiveness of yoga on pain, physical function, and quality of life in older adults with chronic musculoskeletal conditions. Musculoskeletal Care. 2022 Mar;20(1):47-73.	Chronic spinal pain was not meta-analyzed separately from other conditions.
32.	Devan D. A Review of Current Therapeutic Practice for the Management of Chronic Pain. South African J of Occupational Therapy. 2014;44(1):48-51.	The intervention of interest was not meta-analyzed.

	Review excluded	Reason
33.	Dunlap et al. Ai Chi for Balance, Pain, Functional Mobility, and Quality of Life in Adults: A Scoping Review. J Aquat Phys Ther 2021;29:14–28.	The intervention of interest was not meta-analyzed.
34.	Easwaran K, et al. Effectiveness of Tai Chi for health promotion for adults with health conditions: a scoping review of Meta-analyses. Disabil Rehabil. 2021 Oct;43(21):2978-2989.	Overview of reviews.
35.	Elwy AR, et al. A systematic scoping review of complementary and alternative medicine mind and body practices to improve the health of veterans and military personnel. Med Care. 2014 Dec;52(12 Suppl 5):S70-82.	No chronic spinal pain.
36.	Ernst E, et al. How effective is yoga? A concise overview of systematic reviews. Focus on Alternative and Complementary Therapies. 2010;15(4):274-79.	Overview of reviews.
37.	Ferreira et al. Can We Explain Heterogeneity Among Randomized Clinical Trials of Exercise for Chronic Back Pain? A Meta-Regression Analysis of Randomized Controlled Trials. Physical Therapy. 2010;90(10):1383-403.	The intervention of interest was not meta-analyzed separately from other interventions.
38.	Ferro Moura Franco K, et al. Prescription of exercises for the treatment of chronic pain along the continuum of nociplastic pain: A systematic review with meta-analysis. Eur J Pain. 2021 Jan;25(1):51-70.	The intervention of interest was not meta-analyzed separately from other interventions (Tai chi and qigong were mixed in the same meta-analysis).
39.	Forsman AK, et al. Psychosocial interventions for the promotion of mental health and the prevention of depression among older adults. Health Promot Int. 2011 Dec;26 Suppl 1:i85-107.	No chronic spinal pain.
40.	Frutiger M, et al. Systematic Review and Meta-Analysis Suggest Strength Training and Workplace Modifications May Reduce Neck Pain in Office Workers. Pain Pract. 2021 Jan;21(1):100-131.	No intervention of interest.
41.	Geneen LJ, et al. Physical activity and exercise for chronic pain in adults: an overview of Cochrane Reviews. Cochrane Database Syst Rev. 2017 Jan 14;1(1):CD011279.	Overview of reviews.
42.	Ghazi C, et al. Social cognitive or learning theory use to improve self-efficacy in musculoskeletal rehabilitation: A systematic review and meta-analysis. Physiother Theory Pract. 2018 Jul;34(7):495-504.	The intervention of interest was not meta-analyzed separately from other interventions.
43.	Gibbs JC, et al. Exercise for improving outcomes after osteoporotic vertebral fracture. Cochrane Database Syst Rev. 2019 Jul 5;7(7):CD008618.	No intervention of interest.

	Review excluded	Reason
44.	Girard J, et al. The effects of qigong on neck pain: A systematic review. Complement Ther Clin Pract. 2019 Feb;34:23-29.	The intervention of interest was not meta-analyzed.
45.	Goode AP, et al. An evidence map of yoga for low back pain. Complement Ther Med. 2016 Apr;25:170-7.	Overview of reviews.
46.	Griffiths GS, et al. Occupational Therapy Interventions for Persistent Pain: A Systematic Review. New Zealand Journal of Occupational Therapy. 2021:68(1):15-22.	The intervention of interest was not meta-analyzed.
47.	Gross AR, et al. Exercises for mechanical neck disorders: A Cochrane review update. Man Ther. 2016 Aug;24:25-45.	Abbreviated report of the included Cochrane reviews.
48.	Guo Y, et al. Beneficial Effects of Qigong Wuqinxi in the Improvement of Health Condition, Prevention, and Treatment of Chronic Diseases: Evidence from a Systematic Review. Evid Based Complement Alternat Med. 2018 Oct 24;2018:3235950.	The intervention of interest was not meta-analyzed.
49.	Gustafsson S, et al. Multi-component health promotion and disease prevention for community-dwelling frail elderly persons: a systematic review. Eur J Ageing. 2009 Oct 17;6(4):315.	The intervention of interest was not meta-analyzed.
50.	Gutke A, et al. Treatments for pregnancy-related lumbopelvic pain: a systematic review of physiotherapy modalities. Acta Obstet Gynecol Scand. 2015 Nov;94(11):1156-67.	Pregnancy-related low back pain.
51.	Hanada EY. Efficacy of rehabilitative therapy in regional musculoskeletal conditions. Best Pract Res Clin Rheumatol. 2003 Feb;17(1):151-66.	The intervention of interest was not meta-analyzed.
52.	Hawk C, et al. Best Practices for Chiropractic Management of Patients with Chronic Musculoskeletal Pain: A Clinical Practice Guideline. J Altern Complement Med. 2020 Oct;26(10):884-901.	Overview of reviews.
53.	Hayden JA, et al. Exercise therapy for treatment of non-specific low back pain. Cochrane Database Syst Rev. 2005 Jul 20;(3):CD000335.	The intervention of interest was not meta-analyzed.
54.	Hayden JA, et al. Exercise therapy for chronic low back pain. Cochrane Database Syst Rev. 2021 Sep 28;9(9):CD009790.	The intervention of interest was not meta-analyzed separately from other interventions.
55.	Hegmann KT, et al. Non-Invasive and Minimally Invasive Management of Low Back Disorders. J Occup Environ Med. 2020 Mar;62(3):e111-e138.	The intervention of interest was not meta-analyzed.

	Review excluded	Reason
56.	Hidalgo B, et al. The efficacy of manual therapy and exercise for treating non-specific neck pain: A systematic review. J Back Musculoskelet Rehabil. 2017 Nov 6;30(6):1149-1169.	No intervention of interest.
57.	Hill C. Is yoga an effective treatment in the management of patients with chronic low back pain compared with other care modalities - a systematic review. J Complement Integr Med. 2013 May 7;10:/j/jcim.2013.10.issue-1/jcim-2012-0007/jcim-2012-0007.xml.	The intervention of interest was not meta-analyzed.
58.	Howe TE, et al. Exercise for preventing and treating osteoporosis in postmenopausal women. Cochrane Database Syst Rev. 2011 Jul 6;(7):CD000333.	No outcome of interest.
59.	Hu X, et al. Effects of exercise therapy for pregnancy-related low back pain and pelvic pain: A protocol for systematic review and meta-analysis. Medicine (Baltimore). 2020 Jan;99(3):e17318.	Review protocol.
60.	Huang YC, et al. Culturally-tailored interventions for chronic disease self-management among Chinese Americans: a systematic review. Ethn Health. 2020 Apr;25(3):465-484.	The intervention of interest was not meta-analyzed.
61.	Jasmin M, et al. Effectiveness of Yoga Intervention for Chronic Neck Pain: A Systematic Literature Review. International Journal of Caring Sciences. 2019;12(2):1088-1096.	The intervention of interest was not meta-analyzed.
62.	Jones K, et al. Interventions for promoting physical activity in people with neuromuscular disease. Cochrane Database Syst Rev. 2021 May 24;5(5):CD013544.	No chronic spinal pain.
63.	Joypaul S, et al. Multi-disciplinary interventions for chronic pain involving education: A systematic review. PLoS One. 2019 Oct 2;14(10):e0223306.	The intervention of interest was not meta-analyzed.
64.	Kader M, et al. Physical activity and exercise during pregnancy. European Journal of Physiotherapy. 2014;16:2-9.	Pregnancy-related low back pain.
65.	Kay TM, et al. Exercises for mechanical neck disorders. Cochrane Database Syst Rev. 2012 Aug 15;(8):CD004250.	Previous versions of included Cochrane Reviews
66.	Kelley GA, et al. Exercise and bone mineral density in men: a meta-analysis of randomized controlled trials. Bone. 2013 Mar;53(1):103-11.	No chronic spinal pain.
67.	Kelley GA, et al. Meditative Movement Therapies and Health-Related Quality-of-Life in Adults: A Systematic Review of Meta-Analyses. PLoS One. 2015 Jun 8;10(6):e0129181.	Overview of reviews.
68.	Ketenci A, et al. Pharmacological and non-pharmacological treatment approaches to chronic lumbar back pain. Turk J Phys Med Rehabil. 2021 Mar 4;67(1):1-10.	The intervention of interest was not meta-analyzed.

	Review excluded	Reason
69.	Kim SD. Effects of yoga on chronic neck pain: a systematic review of randomized controlled trials. J Phys Ther Sci. 2016 Jul;28(7):2171-4.	The intervention of interest was not meta-analyzed.
70.	Kim KV, et al. Effect of yoga on health-related outcomes in people at risk of fractures: a systematic review. Appl Physiol Nutr Metab. 2022 Mar;47(3):215-226.	The intervention of interest was not meta-analyzed.
71.	Kinser PA, et al. Physical Activity and Yoga-Based Approaches for Pregnancy-Related Low Back and Pelvic Pain. J Obstet Gynecol Neonatal Nurs. 2017 May-Jun;46(3):334-346.	Pregnancy-related low back pain.
72.	Koukoulithras I Sr, et al. The Effectiveness of Non-Pharmaceutical Interventions Upon Pregnancy-Related Low Back Pain: A Systematic Review and Meta-Analysis. Cureus. 2021 Jan 30;13(1):e13011.	Pregnancy-related low back pain.
73.	Larmer PJ, et al. Hydrotherapy outcome measures for people with arthritis: A systematic review. New Zealand Journal of Physiotherapy. 2014;42(2):54-67.	No chronic spinal pain.
74.	Leaver AM, et al. Conservative interventions provide short-term relief for non-specific neck pain: a systematic review. J Physiother. 2010;56(2):73-85.	No intervention of interest.
75.	Lee MS, et al. Tai chi for osteoporosis: a systematic review. Osteoporos Int. 2008 Feb;19(2):139-46.	No chronic spinal pain.
76.	Lee MS, et al. Internal qigong for pain conditions: a systematic review. J Pain. 2009 Nov;10(11):1121-1127.e14.	The intervention of interest was not meta-analyzed.
77.	Lee C, et al. An analysis of the various chronic pain conditions captured in a systematic review of active self-care complementary and integrative medicine therapies for the management of chronic pain symptoms. Pain Med. 2014 Apr;15 Suppl 1:S96-103.	The intervention of interest was not meta-analyzed.
78.	Lemieux J, et al. Comparing the effectiveness of group-based exercise to other non-pharmacological interventions for chronic low back pain: A systematic review. PLoS One. 2020 Dec 30;15(12):e0244588.	The intervention of interest was not meta-analyzed.
79.	Leung KW, et al. Mind-Body Health Benefits of Traditional Chinese Qigong on Women: A Systematic Review of Randomized Controlled Trials. Evid Based Complement Alternat Med. 2021 Sep 14;2021:7443498.	The intervention of interest was not meta-analyzed.

	Review excluded	Reason
80.	Lewis A, et al. Are physiotherapy exercises effective in reducing chronic low back pain? Physical Therapy Reviews. 2008;13(1):37-44.	The intervention of interest was not meta-analyzed.
81.	Lewis GN, et al. How Have Chronic Pain Management Programs Progressed? A Mapping Review. Pain Pract. 2019 Sep;19(7):767-784.	The intervention of interest was not meta-analyzed.
82.	Li G, et al. Effects of Tai Chi on health related quality of life in patients with chronic conditions: a systematic review of randomized controlled trials. Complement Ther Med. 2014 Aug;22(4):743-55.	The intervention of interest was not meta-analyzed.
83.	Li H, et al. Baduanjin exercise for low back pain: A systematic review and meta-analysis. Complement Ther Med. 2019 Apr;43:109-116.	Chronic spinal pain was not meta-analyzed separately from other conditions (some trials did not report the phase of low back pain).
84.	Li G, et al. The effect of whole body vibration on health-related quality of life in patients with chronic conditions: a systematic review. Qual Life Res. 2019 Nov;28(11):2859-2870.	No intervention of interest.
85.	Li Z, et al. Therapeutic Effects of Traditional Chinese Exercises on Musculoskeletal Pain: A Systematic Review and Meta-Analysis. Pain Res Manag. 2021 May 10;2021:5584997.	The intervention of interest was not meta-analyzed separately from other interventions (Tai chi and qigong were mixed in the same meta-analysis for low back pain).
86.	Liddle SD, et al. Interventions for preventing and treating low-back and pelvic pain during pregnancy. Cochrane Database Syst Rev. 2015 Sep 30;2015(9):CD001139.	Pregnancy-related low back pain.
87.	Lin HT, et al. A Scoping Review of The Efficacy of Virtual Reality and Exergaming on Patients of Musculoskeletal System Disorder. J Clin Med. 2019 Jun 4;8(6):791.	The intervention of interest was not meta-analyzed.
88.	Liu B, et al. The efficacy and safety of Health Qigong for ankylosing spondylitis: Protocol for a systematic review and meta-analysis. Medicine (Baltimore). 2020 Jan;99(3):e18734.	Review protocol.
89.	Liu W, et al. Effects of traditional qigong exercise on ankylosing spondylitis: a protocol for systematic reviews and meta-analysis. BMJ Open. 2021 Apr 21;11(4):e046188.	Review protocol.
90.	Lorenc A, et al. Scoping review of systematic reviews of complementary medicine for musculoskeletal and mental health conditions. BMJ Open. 2018 Oct 15;8(10):e020222.	Overview of reviews.

	Review excluded	Reason
91.	Macedo LG, et al. Motor control exercise for persistent, nonspecific low back pain: a systematic review. Phys Ther. 2009 Jan;89(1):9-25.	No intervention of interest.
92.	Macedo LG, et al. Physical therapy interventions for degenerative lumbar spinal stenosis: a systematic review. Phys Ther. 2013 Dec;93(12):1646-60.	No intervention of interest.
93.	Majid SS, et al. Effect of yoga intervention among patients undergoing low back pain treatment: a literature review. Enfermería Clínica. 2020;20(S2):177-81.	The intervention of interest was not meta-analyzed.
94.	Manheimer E, et al. Evidence from the Cochrane Collaboration for Traditional Chinese Medicine therapies. J Altern Complement Med. 2009 Sep;15(9):1001-14.	Overview of reviews.
95.	Marks R. Qigong and Musculoskeletal Pain. Curr Rheumatol Rep. 2019 Nov 16;21(11):59.	The intervention of interest was not meta-analyzed.
96.	Martinez-Calderon J, et al. Which Interventions Enhance Pain Self-efficacy in People With Chronic Musculoskeletal Pain? A Systematic Review With Meta-analysis of Randomized Controlled Trials, Including Over 12 000 Participants. J Orthop Sports Phys Ther. 2020 Aug;50(8):418-430.	The intervention of interest was not meta-analyzed separately from other interventions.
97.	McGregor AH, et al. Rehabilitation following surgery for lumbar spinal stenosis. Cochrane Database Syst Rev. 2013 Dec 9;(12):CD009644.	No intervention of interest.
98.	Meereis Lemos ECW, et al. Influence of strength training and multicomponent training on the functionality of older adults: systematic review and meta-analysis. Rev Bras Cineantropom Hum. 2020;22:e60707.	The intervention of interest was not meta-analyzed separately from other interventions.
99.	Merepeza A. Effects of spinal manipulation versus therapeutic exercise on adults with chronic low back pain: a literature review. J Can Chiropr Assoc. 2014 Dec;58(4):456-66.	No intervention of interest.
100	Mooventhan A. A comprehensive review on scientific evidence-based effects (including adverse effects) of yoga for normal and high-risk pregnancy-related health problems. J Bodyw Mov Ther. 2019 Oct;23(4):721-727.	Pregnancy-related low back pain.
101	Morone NE, et al. Mind-body interventions for chronic pain in older adults: a structured review. Pain Med. 2007 May-Jun;8(4):359-75.	The intervention of interest was not meta-analyzed.
102	Mottola MF, et al. 2019 Canadian guideline for physical activity throughout pregnancy. Br J Sports Med. 2018 Nov;52(21):1339-1346.	Pregnancy-related low back pain.

	Review excluded	Reason
103	Murthy V, et al. An integrative review of complementary and alternative medicine use for back pain: a focus on prevalence, reasons for use, influential factors, self-perceived effectiveness, and communication. Spine J. 2015 Aug 1;15(8):1870-83.	The intervention of interest was not meta-analyzed.
104	Nascimento PRCD, et al. Effectiveness of interventions for non-specific low back pain in older adults. A systematic review and meta-analysis. Physiotherapy. 2019 Jun;105(2):147-162.	One randomized controlled trial of interest was only meta-analyzed.
105	Nelson EA, et al. Systematic review of the efficacy of pre-surgical mind-body based therapies on post-operative outcome measures. Complement Ther Med. 2013 Dec;21(6):697-711.	No intervention of interest.
106	Ng BH, et al. Psychophysiological outcomes of health qigong for chronic conditions: a systematic review. Psychophysiology. 2009 Mar;46(2):257-69.	Chronic spinal pain was not meta-analyzed separately from other conditions.
107	O'Keeffe M, et al. Comparative Effectiveness of Conservative Interventions for Nonspecific Chronic Spinal Pain: Physical, Behavioral/Psychologically Informed, or Combined? A Systematic Review and Meta-Analysis. J Pain. 2016 Jul;17(7):755-74.	The intervention of interest was not meta-analyzed separately from other interventions.
108	Olsen PØ, et al. Effects of resistance training on self-reported disability in older adults with functional limitations or disability - a systematic review and meta-analysis. Eur Rev Aging Phys Act. 2019 Dec 7;16:24.	No chronic spinal pain.
109	Park J, et al. A Narrative Review of Movement-Based Mind-Body Interventions: Effects of Yoga, Tai Chi, and Qigong for Back Pain Patients. Holist Nurs Pract. 2020 Jan/Feb;34(1):3-23.	The intervention of interest was not meta-analyzed.
110	Paul CL, et al. The impact of web-based approaches on psychosocial health in chronic physical and mental health conditions. Health Educ Res. 2013 Jun;28(3):450-71.	No chronic spinal pain.
111	Peek AL, et al. Different forms of exercise for chronic low back pain (PEDro synthesis). Br J Sports Med. 2016 Feb;50(3):188. (This study was based on Searle A, Spink M, Ho A, Chuter V. Exercise interventions for the treatment of chronic low back pain: a systematic review and meta-analysis of randomised controlled trials. Clin Rehabil. 2015 Dec;29(12):1155-67.)	The intervention of interest was not meta-analyzed separately from other interventions.
112	Petersen CM, et al. Proprioception interventions to improve cervical position sense in cervical pathology. International Journal of Therapy and Rehabilitation. 2013;20(3):154-63.	No intervention of interest.
113	Phadsri S, et al. Nonpharmacological Treatment for Supporting Social Participation of Adults with Depression. Occup Ther Int. 2021 Apr 29;2021:8850364.	No chronic spinal pain.

	Review excluded	Reason
114	Posadzki P, et al. Is yoga effective for pain? A systematic review of randomized clinical trials. Complement Ther Med. 2011 Oct;19(5):281-7.	The intervention of interest was not meta-analyzed.
115	Quentin C, et al. Effect of Home Exercise Training in Patients with Nonspecific Low-Back Pain: A Systematic Review and Meta-Analysis. Int J Environ Res Public Health. 2021 Aug 10;18(16):8430.	Data unavailable.
116	Quinn F, et al. Complementary and alternative medicine in the treatment of low back pain: A systematic review. Physical Therapy Reviews. 2006;11:107-16.	The intervention of interest was not meta-analyzed.
117	Ranjeeta A, et al. Physiotherapy Management of Chronic Back Pain: Systematic Literature Review. Indian Journal of Physiotherapy and Occupational Therapy. 2011;5(4):167-70.	The intervention of interest was not meta-analyzed.
118	Regnaux JP, et al. Exercise programmes for ankylosing spondylitis. Cochrane Database Syst Rev. 2019 Oct 2;10(10):CD011321.	No intervention of interest.
119	Reid KF, et al. The Effects of Tai Chi Mind-Body Approach on the Mechanisms of Gulf War Illness: an Umbrella Review. Integr Med Res. 2019 Sep;8(3):167-172.	Overview of reviews.
120	Rothberg S, et al. Complementary therapies in addition to medication for patients with nonchronic, nonradicular low back pain: a systematic review. Am J Emerg Med. 2017 Jan;35(1):55-61.	No chronic spinal pain.
121	Schoutens C, et al. Outcomes of Nonsurgical Treatments for Symptomatic Adult Degenerative Scoliosis: A Systematic Review. Pain Med. 2020 Jun 1;21(6):1263-1275.	The intervention of interest was not meta-analyzed.
122	Sexton BP, et al. To sit or not to sit? A systematic review and meta-analysis of seated exercise for older adults. Australas J Ageing. 2019 Mar;38(1):15-27.	No chronic spinal pain.
123	Sharma M, et al. Yoga as an Alternative and Complementary Treatment for Patients With Low Back Pain: A Systematic Review. Journal of Evidence-Based Complementary & Alternative Medicine. 2013;18(1):23-8.	The intervention of interest was not meta-analyzed.
124	Singh A, et al. Yoga for Neck Pain: A Review. Alternative and Complementary Therapies. 2020;26(5):205-09.	The intervention of interest was not meta-analyzed.
125	Southerst D, et al. Is exercise effective for the management of neck pain and associated disorders or whiplash-associated disorders? A systematic review by the Ontario Protocol for Traffic Injury Management (OPTIMa) Collaboration. Spine J. 2016 Dec;16(12):1503-1523.	The intervention of interest was not meta-analyzed.

	Review excluded	Reason
126	Sun W, et al. Comparative efficacy of 12 non-drug interventions on non-specific chronic low back pain in nurses: A systematic review and network meta-analysis. J Back Musculoskelet Rehabil. 2021;34(4):499-510.	Network meta-analysis.
127	Sutar R, et al. Yoga intervention and functional pain syndromes: a selective review. Int Rev Psychiatry. 2016 Jun;28(3):316-22.	Review with mixed designs (e.g., systematic reviews and trials).
128	Swathi PS, et al. Health and therapeutic benefits of Shatkarma: A narrative review of scientific studies. J Ayurveda Integr Med. 2021 Jan-Mar;12(1):206-212.	The intervention of interest was not meta-analyzed.
129	Tan G, et al. Efficacy of selected complementary and alternative medicine interventions for chronic pain. J Rehabil Res Dev. 2007;44(2):195-222.	The intervention of interest was not meta-analyzed.
130	Toneti BF, et al. Benefits of Qigong as an integrative and complementary practice for health: a systematic review. Rev Lat Am Enfermagem. 2020;28:e3317.	The intervention of interest was not meta-analyzed.
131	Tsang TW, et al. Health benefits of Kung Fu: a systematic review. J Sports Sci. 2008 Oct;26(12):1249-67.	The intervention of interest was not meta-analyzed.
132	Tsang HW, et al. Effects of mindful and non-mindful exercises on people with depression: a systematic review. Br J Clin Psychol. 2008 Sep;47(Pt 3):303-22.	The intervention of interest was not meta-analyzed.
133	Tulloch A, et al. Yoga-based exercise improves health-related quality of life and mental well-being in older people: a systematic review of randomised controlled trials. Age Ageing. 2018 Jul 1;47(4):537-544.	Chronic spinal pain was not meta-analyzed separately from other conditions.
134	van Benten E, et al. Recommendations for physical therapists on the treatment of lumbopelvic pain during pregnancy: a systematic review. J Orthop Sports Phys Ther. 2014 Jul;44(7):464-73, A1-15.	Pregnancy-related low back pain.
135	van der Velde G, et al. Identifying the best treatment among common nonsurgical neck pain treatments: a decision analysis. J Manipulative Physiol Ther. 2009 Feb;32(2 Suppl):S209-18.	No intervention of interest.
136	Wang XQ, et al. A meta-analysis of core stability exercise versus general exercise for chronic low back pain. PLoS One. 2012;7(12):e52082.	The intervention of interest was not meta-analyzed separately from other interventions.

	Review excluded	Reason
137	Wasser JG, et al. Exercise Benefits for Chronic Low Back Pain in Overweight and Obese Individuals. PM R. 2017 Feb;9(2):181-192.	The intervention of interest was not meta-analyzed.
138	Wei X, et al. The potential effect of Wuqinxi exercise for primary osteoporosis: A systematic review and meta-analysis. Maturitas. 2015 Dec;82(4):346-54.	One randomized controlled trial of interest was only meta-analyzed.
139	Wieland LS, et al. Evidence on yoga for health: A bibliometric analysis of systematic reviews. Complement Ther Med. 2021 Aug;60:102746.	Overview of reviews.
140	Wilhelm MP, et al. The Effects of Exercise Dosage on Neck-Related Pain and Disability: A Systematic Review With Meta-analysis. J Orthop Sports Phys Ther. 2020 Nov;50(11):607-621.	The intervention of interest was not meta-analyzed separately from other interventions.
141	Williams ACC, et al. Psychological therapies for the management of chronic pain (excluding headache) in adults. Cochrane Database Syst Rev. 2020 Aug 12;8(8):CD007407.	No intervention of interest.
142	Wollesen B, et al. The effects of cognitive-motor training interventions on executive functions in older people: a systematic review and meta-analysis. Eur Rev Aging Phys Act. 2020 Jul 2;17:9.	No chronic spinal pain.
143	Xie YH, et al. Traditional Chinese Mind and Body Exercises for Neck Pain: A Meta-Analysis of Randomized Controlled Trials. Pain Res Manag. 2021 Oct 1;2021:5426595.	The intervention of interest was not meta-analyzed separately from other interventions (Tai chi and qigong were mixed in the same meta-analysis for pain intensity and quality of life).
144	Yan Y, et al. Exercise vs Conventional Treatment for Treatment of Primary Osteoporosis: A Systematic Review and Meta-Analysis of Randomized Controlled Trials. Orthop Surg. 2021 Jul;13(5):1474-1487.	No chronic spinal pain.
145	Yang GY, et al. Determining the safety and effectiveness of Tai Chi: A critical overview of 210 systematic reviews of controlled clinical trials. 2021. (This is a preprint).	Overview of reviews.
146	Ye J, et al. The Neuroscience of Nonpharmacological Traditional Chinese Therapy (NTCT) for Major Depressive Disorder: A Systematic Review and Meta-Analysis. Evid Based Complement Alternat Med. 2019 May 15;2019:2183403.	No chronic spinal pain.

	Review excluded	Reason
147	Yohannes AM, et al. Management of depression in older people with osteoarthritis: A systematic review. Aging & Mental Health. 2010;14(6):637-51.	The intervention of interest was not meta-analyzed.
148	Yu H, et al. TCM nonpharmacological interventions for chronic low-back pain: A protocol for systematic review and network meta-analysis. Medicine (Baltimore). 2020 Oct 2;99(40):e22547.	Review protocol.
149	Zhang SK, et al. Effects of Low Back Pain Exercises on Pain Symptoms and Activities of Daily Living: A Systematic Review and Meta-Analysis. Percept Mot Skills. 2022 Feb;129(1):63-89.	The intervention of interest was not meta-analyzed separately from other interventions.
150	Zhang YH, et al. Exercise for Neuropathic Pain: A Systematic Review and Expert Consensus. Front Med (Lausanne). 2021 Nov 24;8:756940.	Review with mixed designs (e.g., systematic reviews and trials).
151	Zhao Q, et al. The effectiveness of aquatic physical therapy intervention on disease activity and function of ankylosing spondylitis patients: a meta-analysis. Psychol Health Med. 2020 Aug;25(7):832-843.	No intervention of interest.
152	Zhao J, et al. The effects of sitting Tai Chi on physical and psychosocial health outcomes among individuals with impaired physical mobility: A systematic review and meta-analysis. Int J Nurs Stud. 2021 Jun;118:103911.	No chronic spinal pain.
153	Zhou J, et al. Characteristic of Clinical Studies on Baduanjin during 2000-2019: A Comprehensive Review. Evid Based Complement Alternat Med. 2020 Oct 16;2020:4783915.	Review with mixed designs (e.g., systematic reviews and trials).
154	Zhuang Q, et al. Effect of Yijinjing exercise on cervical spondylosis: A protocol for systematic review. Medicine (Baltimore). 2020 Jul 2;99(27):e20764.	Review protocol.
