# Supplementary material for: The Effects of Mind–Body Exercises on Chronic Spinal Pain Outcomes: A Synthesis Based on 72 Meta-Analyses
Source: Int J Environ Res Public Health. 2022 Sep 23;19(19):12062. doi: 10.3390/ijerph191912062 (PMC9564899; doi:10.3390/ijerph191912062)
Supplement: Supplementary file 1 [file ijerph-19-12062-s001.zip › Supplementary File S3.pdf]

Supplementary File S3. Reasons for excluding potential meta-analyzes from our umbrella review.

Study	Meta-analysis	Reason
Anheyer et al. 2021	Figure 2.A	Two trials in this meta-analysis involved people with nonchronic pain (Kim et al. 2014 and Monro et al. 2015).
Bai et al. 2015	Figure 3.A	Chronic conditions were not separately meta-analyzed.
Li et al. 2019	Figure 3. A: 1.1.1 and 1.1.2	Two quasi-randomized trials were included in both meta-analyzes (Dunleavy et al. 2016 and Kim, 2018).
Nduwimana et al. 2020	Figure 2	A trial in this meta-analysis involved people with non-chronic pain (Kim et al. 2014).
Qin et al. 2019	Figure 2	The presence or absence of chronicity could not be determined in one trial (Qing et al. 2012: this trial was published in Chinese language).
	Figure 3: 1.1.2	
Zhang et al. 2019	Figure 2	Interventions of interest were not separately analyzed from other interventions.
Zou et al. 2019	Figure 3	Interventions of interest were not separately analyzed from other interventions.
