# Supplementary material for: The Effects of Mind–Body Exercises on Chronic Spinal Pain Outcomes: A Synthesis Based on 72 Meta-Analyses
Source: Int J Environ Res Public Health. 2022 Sep 23;19(19):12062. doi: 10.3390/ijerph191912062 (PMC9564899; doi:10.3390/ijerph191912062)
Supplement: Supplementary file 1 [file ijerph-19-12062-s001.zip › Supplementary File S4.pdf]

Supplementary File S4. List of reviews that were evaluated after manual searches and subsequently excluded (n = 28).

Bertozzi L, Gardenghi I, Turoni F, Villafane JH, Capra F, Guccione AA, Pillastrini P. Effect of therapeutic exercise on pain and disability in the management of chronic Nonspecific neck pain: Systematic review and meta-analysis of randomized trials. Physical Therapy 2013;93(8):1026–36.

Chou R, Deyo R, Friedly J, et al. Noninvasive Treatments for Low Back Pain. AHRQ Comparative Effectiveness Reviews. Rockville (MD)2016.

Cramer H, Lauche R, Langhorst J, Dobos G. Are Indian yoga trials more likely to be positive than those from other countries? A systematic review of randomized controlled trials. Contemp Clin Trials. 2015 Mar;41:269-72.

Cramer H, Lauche R, Langhorst J, Dobos G. Is one yoga style better than another? A systematic review of associations of yoga style and conclusions in randomized yoga trials. Complement Ther Med. 2016 Apr;25:178-87.

Furlan AD, Yazdi F, Tsertsvadze A, Gross A, Van Tulder M, et al. (2012) A systematic review and meta-analysis of efficacy, cost-effectiveness, and safety of selected complementary and alternative medicine for neck and low-back pain. Evid Based Complement Alternat Med 2012: 953139

Gomes-Neto M, Lopes JM, Conceição CS, Araujo A, Brasileiro A, Sousa C, Carvalho VO, Arcanjo FL. Stabilization exercise compared to general exercises or manual therapy for the management of low back pain: A systematic review and meta-analysis. Phys Ther Sport. 2017 Jan;23:136-142.

Gordon, R.; Bloxham, S. A systematic review of the effects of exercise and physical activity on non-specific chronic low back pain. Healthcare 2016, 4, 22.

Gross AR, Goldsmith C, Hoving JL, Haines T, Peloso P, Aker P, et al and the Cervical Overview Group. Conservative Management of Mechanical Neck Disorders: A Systematic Review. Journal of Rheumatology 2007;34:1083–102.

Hall A, Maher C, Latimer J, Ferreira M. The effectiveness of Tai Chi for chronic musculoskeletal pain conditions: a systematic review and meta-analysis. Arthritis Rheum. 2009;2009:717–724.

Hayden JA, van Tulder MW, Malmivaara AV, Koes BW. Meta-analysis: exercise therapy for nonspecific low back pain. Ann Intern Med. 2005 May 3;142(9):765-75.

Hayden JA, Wilson MN, Stewart S, Cartwright JL, Smith AO, Riley RD, van Tulder M, Bendix T, Cecchi F, Costa LOP, Dufour N, Ferreira ML, Foster NE, Gudavalli MR, Hartvigsen J, Helmhout P, Kool J, Koumantakis GA, Kovacs FM, Kuukkanen T, Long A, Macedo LG, Machado LAC, Maher CG, Mehling W, Morone G, Peterson T, Rasmussen-Barr E, Ryan CG, Sjögren T, Smeets R, Staal JB, Unsgaard-Tøndel M, Wajswelner H, Yeung EW; Chronic Low Back Pain IPD Meta-Analysis Group. Exercise treatment effect modifiers in persistent low back pain: an individual participant data meta-analysis of 3514 participants from 27 randomised controlled trials. Br J Sports Med. 2020 Nov;54(21):1277-1278.

Kang, J. W., Lee, M. S., Posadzki, P. & Ernst, E. T'ai Chi for the treatment of osteoarthritis: a systematic review and meta-analysis. BMJ Open. 1, e000035; doi: 10.1136/bmjopen-2010-000035 (2011).

Kelly Z. Is yoga an effective treatment for low back pain: a research review. Int J Yoga Therapy 2009; 19: 103–12.

Kim, H.; Reece, J.; Kang, M. Effects of Accumulated Short Bouts of Exercise on Weight and Obesity Indices in Adults: A MetaAnalysis. Am. J. Health Promot. 2020, 34, 96–104.

Lacroix, A.; Hortobágyi, T.; Beurskens, R.; Granacher, U. Effects of Supervised vs. Unsupervised Training Programs on Balance and Muscle Strength in Older Adults: A Systematic Review and Meta-Analysis. Sports Med. 2017, 47, 2341–2361.

Lee C, Crawford C, Schoomaker E, et al. Movement therapies for the self-management of chronic pain symptoms. Pain Med 2014;15 (Suppl 1):S40–53

Murphy, M.H.; Lahart, I.; Carlin, A.; Murtagh, E. The Effects of Continuous Compared to Accumulated Exercise on Health: A Meta-Analytic Review. Sports Med. 2019, 49, 1585–1607.

Ostelo RW, de Vet HC, Waddell G, Kerckhoffs MR, Leffers P, van Tulder M. Rehabilitation following first-time lumbar disc surgery: a systematic review within the framework of the cochrane collaboration. Spine (Phila Pa 1976). 2003 Feb 1;28(3):209-18.

Posadzki P, Ernst E. Yoga for low back pain: a systematic review of randomized clinical trials. Clin Rheumatol. 2011;30:1257–1262.

Richmond H, Hall AM, Copsey B, et al. The effectiveness of cognititve behavioural interventions for non-specific low back pain: a systematic review and meta-analysis. PLoS One. 2015;10:e0134192.

Salt E, Wright C, Kelly S, Dean A. A systematic literature review on the effectiveness of non-invasive therapy for cervicobrachial pain. Manual Therapy 2011;16:53–65.

Searle, A.; Spink, M.; Ho, A.; Chuter, V. Exercise interventions for the treatment of chronic low back pain: A systematic review and meta-analysis of randomised controlled trials. Clin. Rehabil. 2015, 29, 1155–1167.

Sorosky S, Stilp S, Akuthota V. Yoga and pilates in the management of low back pain. Curr Rev Musculoskelet Med. 2008 Mar;1(1):39-47.

Wang C. Tai chi and rheumatic diseases. Rheum Dis Clin North Am. 2011 Feb;37(1):19-32.

Zou, L.; SasaKi, J.; Wang, H.; Xiao, Z.; Fang, Q.; Zhang, M. A Systematic Review and Meta-Analysis Baduanjin Qigong for Health Benefits: Randomized Controlled Trials. Evid. Based Complement. Altern. Med. 2017, 2017.

Zou, L.; Sasaki, J.E.; Wei, G.-X.; Huang, T.; Yeung, A.S.; Neto, O.B.; Chen, K.W.; Hui, S.-C. Effects of mind–body exercises (Tai Chi/Yoga) on heart rate variability parameters and perceived stress: A systematic review with meta-analysis of randomized controlled trials. J. Clin. Med. 2018, 7, 404.

Zou L, Yeung A, Quan X, et al. A systematic review and meta-analysis of mindfulness-based (baduanjin) exercise for alleviating musculoskeletal pain and improving sleep quality in people with chronic diseases. Int J Environ Res Public Health 2018;15:206.

Zou, L.; Wang, C.; Yeung, A.; Liu, Y.; Pan, Z. A Review Study on the beneficial effects of baduanjin. J. Altern. Complement. Med. 2018, 24, 324–335.
