# Supplementary material for: The Effects of Mind–Body Exercises on Chronic Spinal Pain Outcomes: A Synthesis Based on 72 Meta-Analyses
Source: Int J Environ Res Public Health. 2022 Sep 23;19(19):12062. doi: 10.3390/ijerph191912062 (PMC9564899; doi:10.3390/ijerph191912062)
Supplement: Supplementary file 1 [file ijerph-19-12062-s001.zip › Supplementary File S5.pdf]

Supplementary File S5. Citation matrices and corrected covered area (CCA) calculations.

Qigong

Randomized controlled trials		Reviews
1.	Blödt S, et al. Qigong versus exercise therapy for chronic low back pain in adults--a randomized controlled non-inferiority trial. Eur J Pain. 2015;19(1):123-31.	- Nduwimana et al. 2020<br>- Zhang et al. 2019<br>- Zou et al. 2019
2.	Phattharasupharerk S, et al. Effects of Qigong practice in office workers with chronic non-specific low back pain: A randomized control trial. J Bodyw Mov Ther. 2019;23(2):375-381.	- Nduwimana et al. 2020<br>- Zhang et al. 2019
3.	Rendant D, et al. Qigong versus exercise versus no therapy for patients with chronic neck pain: a randomized controlled trial. Spine (Phila Pa 1976). 2011;36(6):419-27.	- Bai et al. 2015<br>- Gross et al. 2015<br>- Yuan et al. 2015
4.	Teut M, et al. Qigong or Yoga Versus No Intervention in Older Adults With Chronic Low Back Pain-A Randomized Controlled Trial. J Pain. 2016;17(7):796-805.	- Nduwimana et al. 2020<br>- Zhang et al. 2019<br>- Zou et al. 2019
5.	von Trott P, et al. Qigong and exercise therapy for elderly patients with chronic neck pain (QIBANE): a randomized controlled study. J Pain. 2009;10(5):501-8.	- Bai et al. 2015<br>- Gross et al. 2015<br>- Yuan et al. 2015

$$CCA = \frac{N-r}{rc-r} = \frac{14-5}{30-5} = \frac{9}{25} \; 0{,}36 = 36\%$$

Note: *N* is the number of included publications (including double counting) in the available evidence synthesis (this is the sum of the ticked boxes in the citation matrix); where *r* is the number of rows (number of index publications), and *c* is the number of columns (number of reviews).

Tai Chi

Randomized controlled trials		Reviews
1	Hall AM, et al. Tai chi exercise for treatment of pain and disability in people with persistent low back pain: a randomized controlled trial. Arthritis Care Res (Hoboken). 2011;63(11):1576-83.	- Hall et al. 2017<br>- Kong et al. 2016<br>- Nduwimana et al. 2020<br>- Qin et al. 2019<br>- Zou et al. 2019
2	Liu J, et al. Chen-Style Tai Chi for Individuals (Aged 50 Years Old or Above) with Chronic Non-Specific Low Back Pain: A Randomized Controlled Trial. Int J Environ Res Public Health. 2019;16(3):517.	- Qin et al. 2019<br>- Zou et al. 2019
3	Qing GM. Study on the curative effective of Taiji boxing in treatment of lumbar disc herniation. Mod Preventi Med. 2012;39:4170–4172. (Chinese) (2012)	- Kong et al. 2016
4	Song H, et al. A study on effect of Taijiquan on lumbar disc protrusion. J Beijing Spt Univ. 2008;3:627–629. (Chinese)	- Kong et al. 2016
5	Tong X, et al. The effects of Tai Chi Tui Shou for non-specific low back pain. Chinese Manipul Rehabil Med. 2016;7:25–7.	- Qin et al. 2019

6.	Weifen W, et al. Effectiveness of tai chi practice for nonspecific chronic low back pain on retired athletes: a randomized controlled study. J Musculoskeletal Pain. 2013;21(1):37–45.	- Hall et al. 2017<br>- Nduwimana et al. 2020

$$CCA = \frac{N-r}{rc-r} = \frac{12-6}{30-6} = \frac{6}{24}\ 0{,}25 = 25\%$$

Note: *N* is the number of included publications (including double counting) in the available evidence synthesis (this is the sum of the ticked boxes in the citation matrix); where *r* is the number of rows (number of index publications), and *c* is the number of columns (number of reviews).

Yoga

Randomized controlled trials		Reviews
1	Aboagye E, et al. Cost-effectiveness of early interventions for non-specific low back pain: a randomized controlled study investigating medical yoga, exercise therapy and self-care advice. J Rehabil Med. 2015;47(2):167-73.	- Anheyer et al. 2021
2	Brämberg EB, et al. Effects of yoga, strength training and advice on back pain: a randomized controlled trial. BMC Musculoskelet Disord. 2017;18(1):132.	- Anheyer et al. 2021<br>- Skelly et al. 2020
3	Chong Y, et al. Effect of hatha yoga on rehabilitation of cervical spondylopathy. J Beijing Sport Univ 2014;37:71–5. [In Chinese].	- Li et al. 2019
4	Cox H, et al. A randomised controlled trial of yoga for the treatment of chronic low back pain: results of a pilot study. Complement Ther Clin Pract. 2010;16(4):187-93.	- Anheyer et al. 2021<br>- Cramer et al. 2013<br>- Kim 2020<br>- Wieland et al. 2017

		- Zhu et al. 2020<br>- Zou et al. 2019
5	Cramer H, et al. Randomized-controlled trial comparing yoga and home-based exercise for chronic neck pain. Clin J Pain. 2013;29(3):216-23.	- Cramer et al. 2017<br>- Li et al. 2019
6	Demirel A, et al. Stabilization exercise versus yoga exercise in non-specific low back pain: Pain, disability, quality of life, performance: a randomized controlled trial. Complement Ther Clin Pract. 2019;35:102-108.	- Anheyer et al. 2021
7.	Groessl EJ, et al. Yoga for Military Veterans with Chronic Low Back Pain: A Randomized Clinical Trial. Am J Prev Med. 2017;53(5):599-608.	- Anheyer et al. 2021<br>- Skelly et al. 2020<br>- Zhu et al. 2020
8.	Groessl EJ, et al. Secondary Outcomes from a Randomized Controlled Trial of Yoga for Veterans with Chronic Low-Back Pain. Int J Yoga Therap. 2020;30(1):69-76. doi: 10.17761/2020-D-19-00036	- Anheyer et al. 2021
9.	Highland KB, et al. Benefits of the Restorative Exercise and Strength Training for Operational Resilience and Excellence Yoga Program for Chronic Low Back Pain in Service Members: A Pilot Randomized Controlled Trial. Arch Phys Med Rehabil. 2018;99(1):91-98.	- Anheyer et al. 2021<br>- Nduwimana et al. 2020<br>- Skelly et al. 2020
10.	Jacobs BP, et al. Feasibility of conducting a clinical trial on Hatha yoga for chronic low back pain: methodological lessons. Altern Ther Health Med. 2004;10(2):80-3. Erratum in: Altern Ther Health Med. 2004;10(3)48.	- Anheyer et al. 2021<br>- Kim 2020<br>- Wieland et al. 2017<br>- Zhu et al. 2020
11.	Jeitler M, et al. Effectiveness of jyoti meditation for patients with chronic neck pain and psychological distress--a randomized controlled clinical trial. J Pain. 2015;16(1):77-86.	- Li et al. 2019
12.	Kuvačić G, et al. Effectiveness of yoga and educational intervention on disability, anxiety, depression, and pain in people with CLBP: A randomized controlled trial. Complement Ther Clin Pract. 2018;31:262-267	- Zhu et al. 2020
13.	Michalsen A, et al. Yoga for chronic neck pain: a pilot randomized controlled clinical trial. J Pain. 2012;13(11):1122-30	- Cramer et al. 2017<br>- Li et al. 2019
14.	Michalsen A, et al. Yoga, Eurythmy Therapy and Standard Physiotherapy (YES-Trial) for Patients With Chronic Non-specific Low Back Pain: A Three-Armed Randomized Controlled Trial. J Pain. 2021;22(10):1233-1245	- Anheyer et al. 2021
15.	Nambi GS, et al. Changes in pain intensity and health related quality of life with Iyengar yoga in nonspecific chronic low back pain: A randomized controlled study. Int J Yoga. 2014;7(1):48-53	- Anheyer et al. 2021<br>- Nduwimana et al. 2020<br>- Skelly et al. 2020<br>- Zhu et al. 2020

16.	Neyaz O, et al. Effectiveness of Hatha Yoga Versus Conventional Therapeutic Exercises for Chronic Nonspecific Low-Back Pain. J Altern Complement Med. 2019;25(9):938-945	- Zhu et al. 2020
17.	Patil NJ, et al. A Randomized Trial Comparing Effect of Yoga and Exercises on Quality of Life in among nursing population with Chronic Low Back Pain. Int J Yoga. 2018;11(3):208-214	- Anheyer et al. 2021
18.	Rajalaxmi V, et al. To analyse the effectiveness of yoga, pilates and Tai Chi exercise for chronic mechanical neck pain—a randomized controlled trail. Biomedicine 2018;38:156–60.	- Li et al. 2019
19.	Saper RB, et al. Yoga for chronic low back pain in a predominantly minority population: a pilot randomized controlled trial. Altern Ther Health Med. 2009;15(6):18-27	- Anheyer et al. 2021<br>- Cramer et al. 2013<br>- Holtzman et al. 2013<br>- Kim 2020<br>- Nduwimana et al. 2020<br>- Ward et al. 2013<br>- Wieland et al. 2017<br>- Zhu et al. 2020<br>- Zou et al. 2019
20.	Saper RB, et al. Yoga, Physical Therapy, or Education for Chronic Low Back Pain: A Randomized Noninferiority Trial. Ann Intern Med. 2017;167(2):85-94	- Anheyer et al. 2021<br>- Skelly et al. 2020<br>- Zhu et al. 2020<br>- Zou et al. 2019
21.	Sherman KJ, et al. Comparing yoga, exercise, and a self-care book for chronic low back pain: a randomized, controlled trial. Ann Intern Med. 2005;143(12):849-56	- Holtzman et al. 2013<br>- Skelly et al. 2020<br>- Slade et al. 2007<br>- Ward et al. 2013<br>- Zhu et al. 2020
22.	Sherman KJ, et al. A randomized trial comparing yoga, stretching, and a self-care book for chronic low back pain. Arch Intern Med. 2011;171(22):2019-26	- Anheyer et al. 2021<br>- Cramer et al. 2013<br>- Holtzman et al. 2013<br>- Kim 2020<br>- Skelly et al. 2020<br>- Ward et al. 2013<br>- Zhu et al. 2020

		- Zou et al. 2019
23.	Tekur P, et al. Effect of short-term intensive yoga program on pain, functional disability and spinal flexibility in chronic low back pain: a randomized control study. J Altern Complement Med. 2008;14(6):637-44	- Cramer et al. 2013<br>- Holtzman et al. 2013<br>- Zhu et al. 2020
24.	Tekur P, et al. Effect of yoga on quality of life of CLBP patients: A randomized control study. Int J Yoga. 2010;3(1):10-7	- Anheyer et al. 2021
25.	Tekur P, et al. A comprehensive yoga programs improves pain, anxiety and depression in chronic low back pain patients more than exercise: an RCT. Complement Ther Med. 2012;20(3):107-18	- Zhu et al. 2020
26.	Telles S, et al. A Randomized Controlled Trial to Assess Pain and Magnetic Resonance Imaging-Based (MRI-Based) Structural Spine Changes in Low Back Pain Patients After Yoga Practice. Med Sci Monit. 2016;22:3228-47	- Kim 2020
27.	Teut M, et al. Qigong or Yoga Versus No Intervention in Older Adults With Chronic Low Back Pain-A Randomized Controlled Trial. J Pain. 2016;17(7):796-805	- Anheyer et al. 2021<br>- Zhu et al. 2020<br>- Zou et al. 2019
28.	Tilbrook HE, et al. Yoga for chronic low back pain: a randomized trial. Ann Intern Med. 2011;155(9):569-78	- Anheyer et al. 2021<br>- Cramer et al. 2013<br>- Kim 2020<br>- Wieland et al. 2017<br>- Zhu et al. 2020<br>- Zou et al. 2019
29.	Uluğ N, et al. Effects of Pilates and yoga in patients with chronic neck pain: A sonographic study. J Rehabil Med. 2018;50(1):80-85	- Li et al. 2019
30.	Wattamwar RB, et al. Effect of conventional occupational therapy and yoga in chronic low back pain. Indian Journal of Occupational Therapy. 2013; 45(3): 13–20.	- Zhu et al. 2020
31.	Williams KA, et al. Effect of Iyengar yoga therapy for chronic low back pain. Pain. 2005;115(1-2):107-17	- Anheyer et al. 2021<br>- Cramer et al. 2013<br>- Holtzman et al. 2013<br>- Skelly et al. 2020<br>- Slade et al. 2007<br>- Wieland et al. 2017<br>- Zhu et al. 2020<br>- Zou et al. 2019

32.	Williams K, et al. Evaluation of the effectiveness and efficacy of Iyengar yoga therapy on chronic low back pain. Spine (Phila Pa 1976). 2009;34(19):2066-76	- Anheyer et al. 2021<br>- Cramer et al. 2013<br>- Holtzman et al. 2013<br>- Nduwimana et al. 2020<br>- Skelly et al. 2020<br>- Ward et al. 2013<br>- Wieland et al. 2017<br>- Zhu et al. 2020<br>- Zou et al. 2019
33.	Yogitha B, et al. Complimentary effect of yogic sound resonance relaxation technique in patients with common neck pain. Int J Yoga. 2010;3(1):18-25	- Cramer et al. 2017<br>- Li et al. 2019

Note: Cramer et al. 2013 considered Tekur et al. 2008 and 2010 as the same study.

$$CCA = \frac{N-r}{rc-r} = \frac{98-33}{429-33} = \frac{65}{396}\ 0{,}16 = 16\%$$

Note: *N* is the number of included publications (including double counting) in the available evidence synthesis (this is the sum of the ticked boxes in the citation matrix); where *r* is the number of rows (number of index publications), and *c* is the number of columns (number of reviews).
